# Multi-objective and multi constrained task scheduling framework for computational grids

**DOI:** 10.1038/s41598-024-56957-8

**Published:** 2024-03-19

**Authors:** Sujay N. Hegde, D. B. Srinivas, M. A. Rajan, Sita Rani, Aman Kataria, Hong Min

**Affiliations:** 1 University of California USA, Irvine, CA USA; 2 Nitte Meenakshi Institute of Technology, Bengaluru, Karnataka 560064 India; 3 TCS Research and Innovation, Bengaluru, Karnataka India; 4 Guru Nanak Dev Engineering College, Ludhiana, Punjab 141006 India; 5 Amity Institute of Defence Technology, Amity University, Noida, U.P. 201303 India; 6 School of Computing, Gachon University, Seongnam, Republic of Korea

**Keywords:** Grid computing, Direct acyclic graph, Scientific graph, GridSim, TOPSIS, Energy science and technology, Engineering, Mathematics and computing

## Abstract

Grid computing emerged as a powerful computing domain for running large-scale parallel applications. Scheduling computationally intensive parallel applications such as scientific, commercial etc., computational grids is a NP-complete problem. Many researchers have proposed several task scheduling algorithms on grids based on formulating and solving it as an optimization problem with different objective functions such as makespan, cost, energy etc. Further to address the requirements/demands/needs of the users (lesser cost, lower latency etc.) and grid service providers (high utilization and high profitability), a task scheduler needs to be designed based on solving a multi-objective optimization problem due to several trade-offs among the objective functions. In this direction, we propose an efficient multi-objective task scheduling framework to schedule computationally intensive tasks on heterogeneous grid networks. This framework minimizes turnaround time, communication, and execution costs while maximizing grid utilization. We evaluated the performance of our proposed algorithm through experiments conducted on standard, random, and scientific task graphs using the GridSim simulator.

## Introduction

Applications with high computational and data demands, such as climate modelling, drug discovery, genomics, bioinformatics, financial modelling, data analytics, and healthcare informatics, are fueling the demand for computational grids^1–10^. Computational grids have emerged as powerful computational paradigms, facilitating large-scale, distributed computing through the utilization of interconnected computing and storage resources. The optimal allocation of tasks to resources in computational grids becomes increasingly intricate due to various constraints, including resource heterogeneity, dynamic workload characteristics, system dynamics, and adherence to user Quality of Service (QoS) parameters, such as latency and cost.

Grid service providers typically aim to maximize profits, while users seek to minimize execution costs, communication costs, and turnaround time for their applications. One approach to achieve this is by designing efficient task schedulers to schedule user applications on computational grids. Efficient task schedulers play a crucial role in achieving these objectives, enabling intelligent decisions regarding task allocation and resource management within specified constraints. Despite being an NP-complete problem^11^, designing efficient task scheduling algorithms for computational grids is essential in meeting user-defined QoS requirements.

The design of task scheduling algorithms is based on either single or multi-objective functions. Task scheduling algorithms based on a single objective function are not suitable for scheduling complex real-time applications. Single-objective task scheduling algorithms primarily focus on optimizing a specific objective ( minimizing makespan, cost, energy etc) based on heuristics, metaheuristic algorithms, or mathematical optimization techniques to find near-optimal scheduling sequences. Single objective functions will find the best solution, which corresponds to either minimum or maximum value. However, they often fail to consider other objectives, resulting in imbalanced resource utilization, increased energy consumption etc. These algorithms are based on meta-heuristic algorithms^12^, greedy^13^, fuzzy model^14^, game theory^15^, bio-inspired^16^, and more. However, in real-world applications, it is necessary to take into account several conflicting goals at once. For instance, maximizing resource utilisation, minimizing turnaround time, minimizing task execution cost etc are equally crucial for improving system efficiency. On the other hand task scheduling algorithms based on multi-objective criteria will address these limitations by simultaneously optimizing multiple objectives, offering more robustness for users to prioritize one or more criteria over other and diverse solutions.

**Figure 1:**
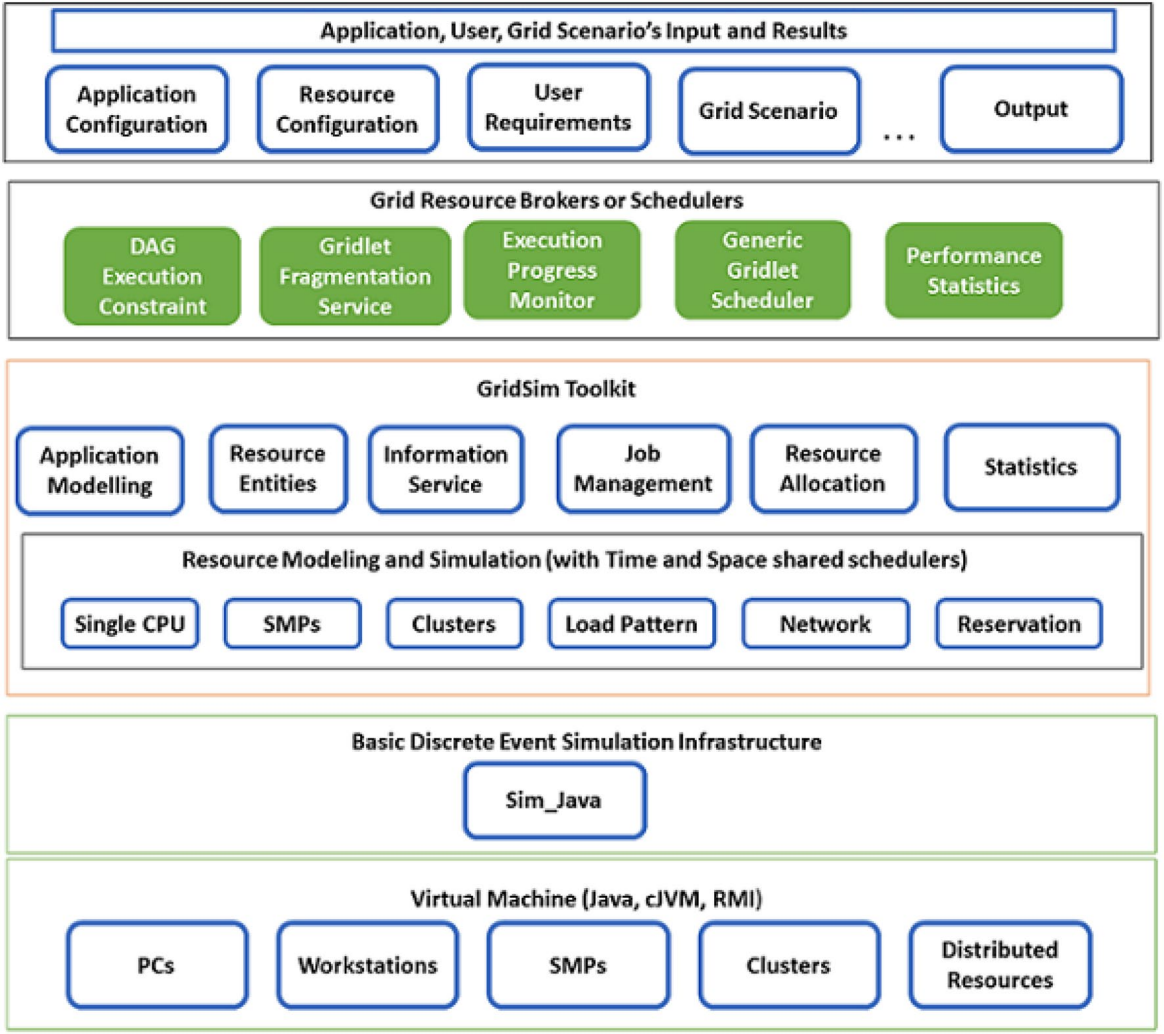
Proposed multi-layer architecture.

Multi-objective function optimization involves optimizing multiple conflicting objectives simultaneously. Common heuristic approaches for multi-objective task scheduling include the application of genetic algorithms (NSGA, NSGA-II)^17,18^, particle swarm optimization (MOPSO)^19^, simulated annealing(MOSA)^20^, ant colony optimization (MOACO)^21^, and other evolutionary (MOEAs)^22^ etc.These methods leverage principles inspired by natural processes to explore the solution space and find trade-off solutions among conflicting objectives. In our proposed method, heuristics are utilized as general problem-solving strategies, employing intuitive, trial-and-error methods to quickly find effective solutions. This systematic approach is designed to identify the best solution based on a defined objective function or set of criteria. Heuristics serve as rule-of-thumb methods, particularly valuable when an exhaustive search or an exact solution is impractical. The objective of incorporating heuristic approaches into our framework is to strike a balance among competing objectives. This includes minimizing turnaround time, execution cost, and communication cost while maximizing resource utilization. The application of heuristics enables the derivation of practical and computationally efficient solutions, especially in scenarios where finding an optimal solution proves challenging or unfeasible. In this article, we propose a task scheduling algorithm based on multi-objective optimization formulation with different objective functions such as minimising turnaround time (TAT), task execution cost, data communication cost between resources, and maximising grid utilization in a heterogeneous multi-grid environment. The proposed framework is plugged into a gridsim architecture as shown in Fig. 1(green colour). The framework contains five different schedulers namely 1. Greedy scheduler: prioritizes minimizing turnaround time, communication cost, and execution cost while maximizing grid utilization. 2. Greedy communication cost scheduler: minimizes communication cost by distributing tasks across computing resources within a single Grid. 3. Greedy execution cost scheduler: aims to minimize execution cost by scheduling each task on the most suitable subset of computing resources based on their cost-to-performance ratio. 4. Greedy no fragmentation scheduler: task as fragmented and schedule tasks on individual computing resources. 5. Random scheduler: schedules tasks on a random subset of computing resources.

We summarize our contributions as follows:

(1) Formulating a task scheduling framework with multiple objectives. (2) The proposed framework is integrated with Grid-sim (simulator) and performance is evaluated. (3) We applied a Technique for Order Preference by Similarity to Ideal Solution (TOPSIS) to solve the proposed multi-objective optimization for task scheduling.

The rest of this paper is organized as follows. Section "Related work" describes the related work. Section "System model" describes the system model. In Sect. "Formulation of multi-objective optimization for task scheduling", objective functions are formulated for TAT, execution cost, communication cost and grid utilization. The task scheduling algorithm is presented in Sect. "Proposed task scheduling algorithm" In Sect. "Demonstration of the proposed task scheduling algorithm" demonstration of our proposed task scheduling is discussed. In Sect. "Results and discussion" results are discussed. Multi-Objective Decision Making Problem is presented in Sect. "Formulation of the multi-Objective-decision-making problem". Finally, in Sect. "Conclusion and future work" we conclude the paper.

## Related work

In this section, we present a brief discussion on existing multi-objective task scheduling frameworks/algorithms/models etc. A Grid-based Evolutionary Algorithm (GrEA) is proposed in Ref.^23^ to tackle multi-objective optimization issues by utilising the grid-based resource capacity to boost selection pressure in the best direction while maintaining a broad and uniform distribution of choices. A framework is designed to evaluate multi-objective functions (makespan, cost, deadline violation rate, and resource utilization.) for scheduling tasks^24^ based on the Ant Colony Algorithm in Cloud Computing. A new bio-inspired diversity metric, Pure Diversity (PD) is proposed in Ref.^25^ to assess the performance of diversity of multi-objective evolutionary algorithms (MOEAs) for solving Many-objective optimization problems(MaOPs). A MATLAB-based PlatEMO is developed to use it for performing comparative experiments, embedding new algorithms, creating new test problems, and developing performance indicators^26^. This platform includes more than 50 multiobjective evolutionary algorithms and more than 100 multi-objective test problems. Multi-objective particle swarm optimizer(NMPSO) algorithm with a Balanceable Fitness Estimation(BFE) method was designed in Ref.^27^ to tackle many-objective optimization problems( MaOPs). A multi-objective optimization method based on a non-dominated sorting genetic algorithm (NSGA-II) is applied and tested on an IEEE 17-bus test system^28^, which simultaneously minimizes two contradicting objective functions such as voltage deviation at buses and total line loss. A multi-objective charging framework that incorporates a vehicle-to-grid (V2G) strategy to optimally manage the real power dispatch of electric cars. The objective functions minimizing load fluctuation and charging costs related with EVs in residential areas^29^. Partitional Clustering Method (PCM) and Hierarchical Clustering Method (HCM) are used in clustering-based evolutionary algorithms for tackling MaOPs^30^. For determining congestion thresholds in low-voltage (LV) grids, authors in Ref.^31^ used a multi-objective particle swarm optimisation (MOPSO) approach paired with data analytics via affinity propagation clustering. A virtual machine migration method is designed to maximize host release and minimize virtual machine migration is proposed in^32^. Task Scheduling for Deadline and Cost Optimization (DCOTS) is presented in Ref.^33^. This work ensures the fulfilment of user requirements while simultaneously aiming to maximize the profitability for cloud providers. The objective functions for building a multi-objective cloud task scheduling model include^34^ execution time, execution cost, and virtual machine load balancing. Subsequently, the task scheduling problem is addressed using the multi-factor optimization (MFO) technique, and the characteristics of task scheduling are integrated with the multi-objective multi-factor optimization (MO-MFO) algorithm to formulate an assisted optimization task. A Task Scheduling technique^35^ based on a Hybrid Competitive Swarm Optimization Algorithm (HCSOA-TS) within the context of the CC platform. The proposed HCSOA-TS efficiently schedules tasks to maximize resource utilization and overall performance. The construction of a multi-objective task scheduling model for cloud computing^36^, aimed at optimizing cloud computing tasks, utilizes the Cat Swarm Optimization (CSO) model. The task objectives for cloud computing were scrutinized, leading to the formulation of a multi-objective task scheduling model with execution time and system load as key scheduling objectives. Study in Ref.^37^ presents a parallel algorithm for task scheduling, where both the priority assignment to tasks and the construction of the heap are concurrently executed. Authors in Ref.^38^ present edge scheduling stage, tasks are arranged based on the latest start times of their successors instead of their sub-deadlines, with the goal of mitigating lateness in subsequent tasks.

In Grid Computing, the resource optimisation problem is treated as a Multi-Objective Optimisation problem^39^, and PSO is used to search the problem area for possible solutions. To find non-dominated solutions for the multi-objective issue and to optimise and search for the best Grid resources, the Functional Code Sieve algorithm is used. Similarly, various task scheduling algorithms^40–47^ based on multi-objective optimization are studied.

Resource management and task scheduling are intricate operations in computational grids. To manage distributed resources and evaluate scheduling algorithms and their performance with different numbers of resources, a toolkit named GridSim has been proposed. GridSim aids in the mapping of user tasks to grid resources. Several task scheduling algorithms have been simulated using GridSim since its introduction^48–55^.

The Technique for Order of Preference by Similarity to Ideal Solution (TOPSIS) is a method used for multi-criteria decision analysis. It was initially introduced in Refs.^56–58^. A-TOPSIS, presented in Ref.^59^, aims to compare the performance of different algorithms based on mean and standard deviations. This technique calculates the best and worst algorithms based on user-defined parameters. Another method, D-TOPSIS, is presented in Ref.^60^ and is more effective in representing uncertain information compared to other group decision support systems based on the classical TOPSIS method. TOPSIS fuzzy^61^ is a multi-objective decision-making tool used to find a scheduling algorithm that can minimize response time and maximize throughput. In Ref.^62^, the authors propose a method that combines the Heterogeneous Earliest Finish Time (HEFT) algorithm with the TOPSIS method to solve multi-objective problems. Thus, TOPSIS is a valuable decision-making technique because it provides a systematic and structured approach to evaluate and rank alternatives based on multiple criteria, helping end users to make well-justified choices in complex decision scenarios.

## System model

### Task model

The task scheduling framework consists of a task graph, a task scheduler and a grid network. A task graph is an input to a task scheduler and is defined as a Weighted Directed Acyclic Graph (WDAG) $WTG=(T, E)$. where *T* is set of tasks and *E* set of edges which describes the dependency between tasks. The weight $W(T_i)$ is assigned to task $T_i$ represents the size of a $i^{\text {th}}$ task and is expressed as Million Instructions (MI).

### Grid model

Grid network consists of set of grid nodes *G* = $\{G_1, G_2, G_3 ,...,G_m\}$ and they are interconnected by high speed network. Each grid node contains *p* number of heterogeneous processing elements $G_i$ = $\{r_{i1}, r_{i2}, r_{i3},...,r_{ip}\}$ and these processing elements are internally connected by a high-speed communication network. Processing speed / CPU_speed of each processor is represented in terms of Million Instructions Per Second (MIPS). Each computational grid contains a local scheduler, The function of the local scheduler is to manage the execution of a task on a grid resource given by the task scheduler. The local scheduler is also responsible for collecting information about computational resources periodically and communicating with the task scheduler.

### Simulation model

#### GridSim^66^

We have employed a Java-based discrete-event toolkit called GridSim to simulate our multi-objective task scheduling framework. This versatile toolkit offers a comprehensive suite of features for modelling and simulating resources and network connectivity, accommodating various capabilities and configurations. Among its capabilities are primitives for composing applications, information services for resource discovery, and interfaces for task allocation to resources and managing their execution. These capabilities enable us to simulate resource brokers or grid schedulers, facilitating the evaluation of scheduling algorithms’ performance. It’s worth noting that GridSim does not prescribe any specific application model, but in our proposed framework, we have adopted a Directed Acyclic Graph (DAG) as the application model. Within the GridSim environment, individual tasks can exhibit differing processing times and input file sizes. To represent these tasks and their requirements, we utilize Gridlet objects. Each Gridlet encapsulates comprehensive information related to a job, including execution management details such as job length (measured in MIPS), disk I/O operations, input and output file sizes, and the job’s originator. In the context of GridSim, a Processing Element (PE) stands as the smallest computing unit, configurable with varying capacities denoted in Million Instructions per Second (MIPS). Multiple PEs can be combined to construct a machine, and in a similar fashion, machines can be aggregated to form a grid. Grids can allocate Gridlets in either a time-sharing mode (common in single-processor Grids) or a space-sharing mode (typical for multi-processor Grids).

#### Existing GridSim architecture

Proposed multi-layer architecture and abstractions are shown in Fig. 1. The layered structure of this system begins with the foundational run-time machinery, known as the JVM (Java Virtual Machine). This JVM is versatile, catering to both single and multiprocessor systems, including clusters. Moving up to the second layer, we encounter a fundamental discrete-event infrastructure that relies on the interfaces offered by the first layer. This infrastructure is actualized through SimJava, a well-regarded Java library for discrete event simulation. The third layer delves into the simulation of essential grid entities, encompassing resources and information services, among others. Here, the GridSim toolkit employs the discrete event services provided by the underlying infrastructure to simulate these core resource entities. Ascending to the fourth layer, our attention turns to the simulation of resource aggregators, often referred to as grid resource brokers or schedulers. Finally, the fifth and topmost layer is dedicated to application and resource modelling across various scenarios. It harnesses the services furnished by the two lower-level layers to evaluate scheduling strategies, resource management policies, heuristics, and algorithms.

### Life cycle of a GridSim simulation

Prior to commencing a simulation, we establish the resource entities (including PEs, Machines, and Grids) that will be available throughout the simulation. Upon GridSim’s initiation, these resource entities autonomously enroll themselves with the Grid Information Service (GIS) entity by dispatching relevant events.

Furthermore, at the onset of the simulation, a user initiates the process by submitting their job to a Resource Broker. The resource broker plays a pivotal role in the simulation, encompassing several responsibilities. It first employs information services to identify accessible resources for the user. Subsequently, it performs task-to-resource mapping (scheduling), orchestrates the staging of application components and data for processing (deployment), initiates job execution, and ultimately aggregates the results. Beyond these tasks, the resource broker also takes on the crucial role of monitoring and tracking the progress of application execution.

### Our resource broker implementation

All the application models we have explored rely on task inter-dependencies, which are precisely defined using Directed Acyclic Graphs (DAGs). Regrettably, GridSim does not inherently accommodate the execution of tasks that are constrained by these inter-dependencies. In response to this limitation, our Resource Broker implementation extends support for such scenarios by ensuring that the order of task execution adheres to the specified dependency constraints. Our Resource Broker defines a versatile task Scheduler interface, offering seamless integration with various schedulers. This interface serves as a plug-and-play mechanism, enabling the utilization of multiple schedulers introduced in our work (GS, GCPS, GEPS, GNFS), all of which adhere to this common interface. Furthermore, our task scheduling framework introduces an innovative concept called task fragmentation, allowing tasks to be divided for execution across multiple computing resources. To facilitate this, our resource broker incorporates a Gridlet Fragmentation Service. When a gridlet is scheduled to run on more than one Processing Element, it is initially fragmented into multiple smaller virtual gridlets. These virtual gridlets are then individually executed by the allocated Processing Elements. Upon their completion, the Gridlet Fragmentation Service reunites them into the original single gridlet. Another novel concept introduced by our task scheduling framework involves partial dependencies among tasks. However, GridSim does not inherently enable the Resource Broker to monitor task progress during execution. To address this, we have implemented a pinger service within the Resource Broker and individual Processing Elements. This pinger service allows the Broker to stay informed about a gridlet’s execution progress, enabling it to schedule child tasks once a parent task has reached a predefined threshold percentage of execution, as dictated by the parent-child dependency.

Lastly, we have enhanced the Resource Broker with the capability to gather performance statistics, including Turnaround Time, Resource Utilization, Execution Price, and Communication Price. These statistics provide valuable insights into the system’s performance.

## Formulation of multi-objective optimization for task scheduling

We propose task scheduling problem as a multi-objective optimization problem with a goal to minimize TAT, execution price, communication price and maximize grid utilization for precedence constrained task graphs is represented as argmin(*TAT*, *EP*,*CP*, $-GU$).

The objective function for TAT is defined and formulated as shown in Eq. (1).

1 $$\begin{aligned} \ TAT = {{\sum }}_{i=1}^{n} {{ \sum }}_{j=1}^{m}{{ \sum }}_{k=1}^{p[j]}{{X_{ij_{k}}} \times \tau _{{ij}_{k}}} \end{aligned}$$

where $${X_{ij_{k}}}={\left\{ \begin{array}{ll} 1, &{}\quad \text {if the task} {T_{i}} \text {is scheduled on the } jth \text { grid} \\ &{}\quad \text {on its } kth \text { resource } \\ 0, &{}\quad \text {otherwise}. \end{array}\right. }$$

$\tau _{{ij}_{k}}=$ Execution time of Task $T_i$ on *k*’th resource of grid *j*

2 $$\begin{aligned} GU= \frac{\sum _{i=0}^{n} W_{T_i}}{\left( \sum _{j=0}^{m} \sum _{k=0}^{j} W_{jk} \right) \times TAT} \end{aligned}$$

Grid Utilization is formulated in Eq. (2).

3 $$\begin{aligned} \begin{aligned} EP&= \sum _{i=0}^{n} \sum _{j=0}^{m} \sum _{k=0}^{p[j]} \left( {X_{ij_{k}}} \times \tau _{{ij}_{k}} \times Price_{E_{kj}} \right) \end{aligned} \end{aligned}$$

Task execution price and communication price is defined and formulated in Eqs. (3) and  (4) respectively. Rest of the paper used price and cost interchangeably.

4 $$\begin{aligned} \ CP = {\sum }_{i=1}^{n} \left( {M_{i} \atopwithdelims ()2} {MAX_{j=1}^{m}} \left( \tau _{ij} \right) \times Price_C \right) \end{aligned}$$

*Where*

$${\tau _{ij}}={\sum }_{k=1} ^{p[j]} {X_{ijk}}*{\tau _{ijk}}$$

*and*

$${M_{i}}={\sum }_{j=1} ^{m} X_{ij}$$

where $${X_{ij}}={\left\{ \begin{array}{ll} 1, &{} \text {if the task }{T_{i}} \text {is scheduled on} \\ &{} \text {on any machine of Grid} G_j \\ 0, &{} \text {otherwise}. \end{array}\right. }$$

## Proposed task scheduling algorithm

Proposed Multi-Objective task scheduling algorithm is described in algorithm 2. Algorithm generates an optimized schedule sequence (task-id, [grid-ID, machine-ID], execution start-time and end-time) according to multiple objectives (TAT, EC, CC and RU).

Input to the algorithm is number of tasks(*n*), task dependency graph (weighted adjacency matrix *WTG*[1, ..., *n*][1, ..., *n*]), task lengths ($$W_T[1,..., n]$$), number of grids(*m*), number of machines *p*[1, ..., *m*] in each grid, processing capacity of each grid in terms of MIPS ($$W_G[1,..., m])$$, and the user’s objective optimization criteria (See 2 for choices). The algorithm’s output is the optimized task schedule sequence (step 1 and 2). Step 3 generates all possible combinatorial subsets of Grid-Machines that a task can be allocated onto, depending on the user’s objective optimization criteria, as so: If the user criteria is *GS* then this step generates all possible subsets of grid-machines sets. If the user criteria is *GCPS* then it generates combinatorial sets of grid machines with all the machines in each set belonging to the same grid. If the user criteria is *GEPS* then it generates combinatorial sets of grid-machines which offer the lowest task execution price (other Grid-Machines are ignored). Similarly, if the user criteria is *GNFS* then it generates singleton sets of all the individual grid-machines.

The algorithm then executes in a loop (from Step 7) until all the tasks have been scheduled. On every iteration of the loop, the algorithm first identifies (in Step 8) tasks whose parent task dependency constraints have been met and are thus available for scheduling. Step 4 then uses function (5) to select the best task and Grid-Machine combination for scheduling. Steps 11 to 13 append this Task-Grid-Machine allocation to the schedule sequence, and update the information about available Grid Machines and unscheduled tasks. Finally, Steps 14, 15 enter into a blocking wait until one or more Grid-Machines are available, after which, the algorithm enters into another iteration of the Step 7 loop.

**Algorithm 1:**
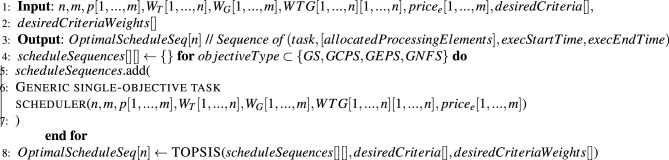
Multi-objective task scheduler.

**Algorithm 2:**
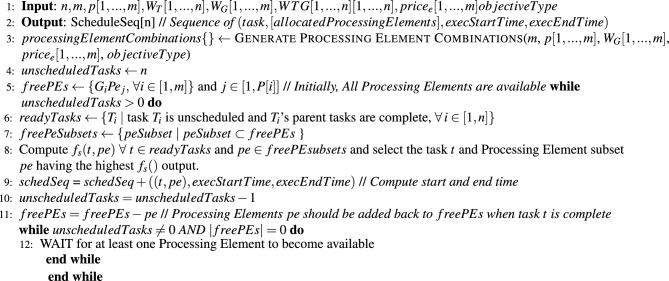
Generic single-objective task scheduler.

**Algorithm 3:**
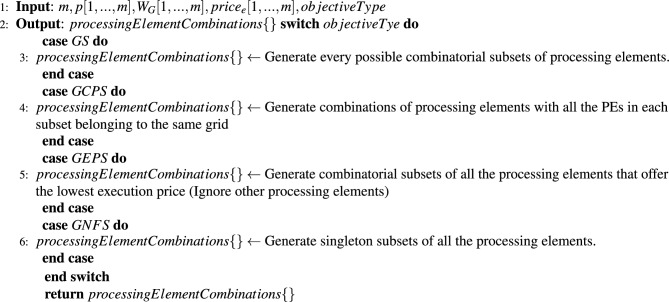
Generate processing element combinations.

Function to determine the preference to schedule a task on a set of GridMachines

5 $$\begin{aligned} f_{s}(T_i, G_jM_k) = \frac{W_{T_i}}{MAX_{i=1}^{n}(W_{T_i})} \times \frac{d^+(T_i)}{MAX_{i=1}^{n}(d^+(T))} \times \frac{W_{G_j}}{MAX_{j=1}^{m}(W_{G_j})} \end{aligned}$$

## Demonstration of the proposed task scheduling algorithm

To enhance comprehension of the proposed algorithm 2, we’ll illustrate its functionality through an example, using concise input parameters. This demonstration will cover four distinct user objective types (*GS*, *GEPS*, *GCPS*, *GNFS*).

**Figure 2:**
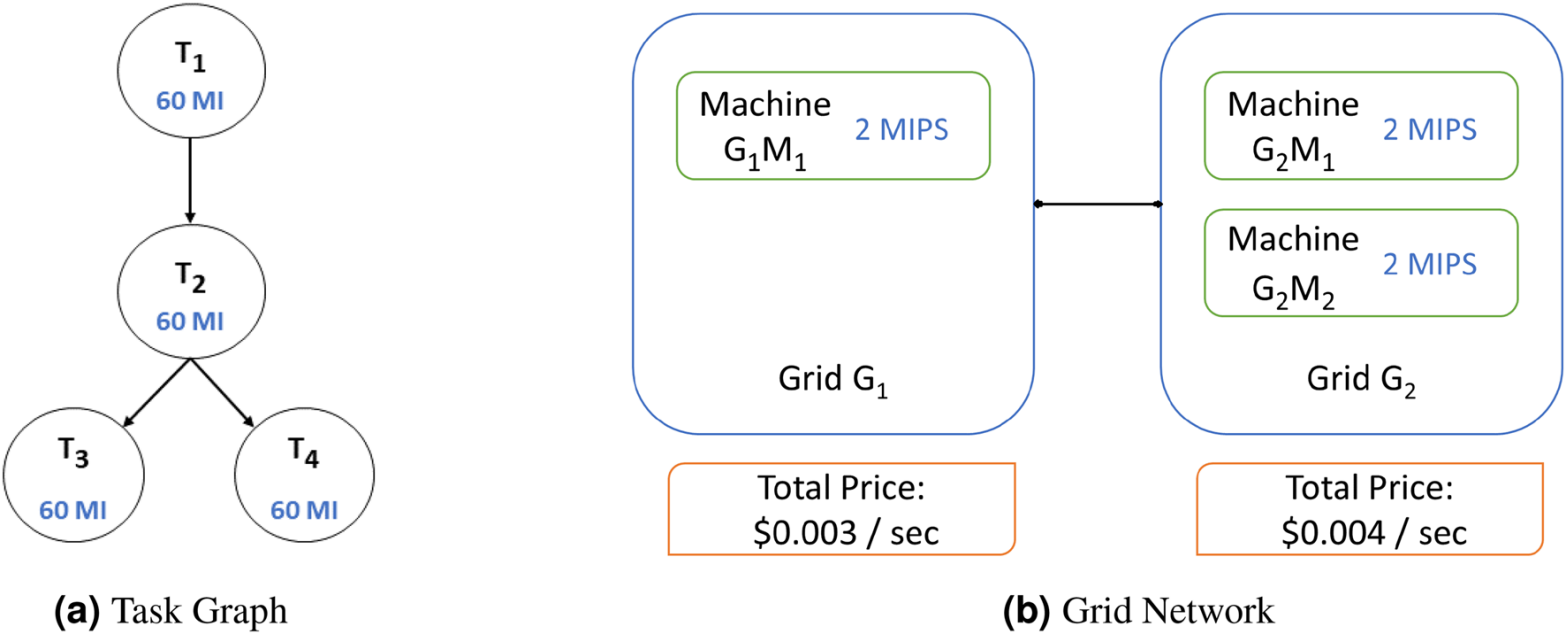
A typical scenario for a proposed scheduling algorithm demonstration.

Consider an application with workload characterized by a task graph comprising four tasks, each task contains 60 million instructions (MI). This task graph is represented as a Directed Acyclic Graph (DAG), as shown in Fig. 2a. Similarly, a grid network, depicted in Fig. 2b, comprises two grids: $G_1$ housing Grid-Machine $G_1M_1$ and $G_2$ hosting Grid-Machines $G_2M_1$ and $G_2M_2$. Each Grid-Machine possesses a processing capacity of 2 million instructions per second (MIPS). These specifications in Table 1, serve as the inputs for Algorithm 2. In the following subsections, we illustrate the iterations executed by the proposed scheduling algorithm and the corresponding helper functions for each distinct *objectiveType*.

**Table 1:** Input parameters to the scheduling algorithm 2.

Input	Value
*n*	4
*m*	2
*p*[1, ..., *m*]	[1, 2]
$W_T[1,..., n]$	[60, 60, 60, 60]
$$W_G[1,..., m]$$	[20, 20]
*WTG*[1, ...*n*][1, ..., *n*]	$$\begin{bmatrix} 0 &{} 100 &{} 0 &{} \\ 0 &{} 0 &{} 100 &{} 0 \\ 0 &{} 0 &{} 100 &{} 100 \\ 0 &{} 0 &{} 0 &{} 0 \\ \end{bmatrix}$$

**Table 2:** Function $f_g()$ to generate possible subsets of Grid machines to allocate tasks to.

*ObjectiveType*	Function $f_g(W_G[1,..., m], p[1,..., m], objectiveType)$
*GS*	Generate all possible combinatorial subset of grid-machines
*GCCS*	Generate combinations of grid-machines with all the machines in each subset belonging to the same grid
*GECS*	Generate combinatorial subsets of all grid-machines that offer the lowest execution price (Ignore other Grid-Machines)
*GNFS*	Generate singleton subsets of all the grid-machines

### Objective type: greedy scheduler

Function $f_g()$ (described in Table 2) generates 7 possible combinations of Grid-Machine subsets to allocate tasks for the Greedy Scheduler *objectiveType*, as illustrated in Table 3.

Function $f_s()$ (described in Eq. (5)) computes a preference matrix for scheduling each task on each of the generated Grid-Machine subsets, as shown in Table 4. Then, Algorithm 2 computes the schedule sequence of task allocations onto Grid-Machines, as shown in Table 5.

**Table 3:** Grid-Machine subsets generated by $f_g()$ for *objectiveType*=*G*.

*GMSubset*	
$$\{G_1M_1\}$$	
$$\{G_2M_1\}$$	
$$\{G_2M_2\}$$	
$$\{G_1M_1, G_2M_1\}$$	
$$\{G_1M_1, G_2M_2\}$$	
$$\{G_2M_1, G_2M_2\}$$	
$$\{G_1M_1, G_2M_1, G_2M_2\}$$

**Table 4:** Function $f_s(task, gmSubset)$ output for *objectiveType*=*G*.

	$T_1$	$T_2$	$T_3$	$T_4$
$\{G_1M_1\}$	20.6	21	20.3	20.3
$\{G_2M_1\}$	20.6	21	20.3	20.3
$\{G_2M_2\}$	20.6	21	20.3	20.3
$\{G_1M_1, G_2M_1\}$	40.6	41	40.3	40.3
$\{G_1M_1, G_2M_2\}$	40.6	41	40.3	40.3
$\{G_2M_1, G_2M_2\}$	40.6	41	40.3	40.3
$\{G_1M_1, G_2M_1, G_2M_2\}$	60.6	61	60.3	60.3

**Table 5:** Schedule sequence of tasks allocations to grid-machines by Greedy scheduler for *objectiveType*=*G*.

Time (s)	Available tasks	freeGMs			$max(f_{s}())$	Generated schedule
		$G_1M_1$	$G_2M_1$	$G_2M_2$		
0	$T_1$	$\checkmark$	$\checkmark$	$\checkmark$	60.6	$T_1 \rightarrow \{G_1M_1, G_2M_1, G_2M_2\}$
1	$T_2$	$\checkmark$	$\checkmark$	$\checkmark$	61.0	$T_2 \rightarrow \{G_1M_1, G_2M_1, G_2M_2\}$
2	$T_3, T_4$	$\checkmark$	$\checkmark$	$\checkmark$	60.33	$T_3 \rightarrow \{G_1M_1, G_2M_1, G_2M_2\}$
3	$T_4$	$\checkmark$	$\checkmark$	$\checkmark$	60.33	$T_4 \rightarrow \{G_1M_1, G_2M_1, G_2M_2\}$
4		$\checkmark$	$\checkmark$	$\checkmark$		Complete; $TAT = 4s$

### Objective type: greedy communication price scheduler

Function $f_g()$ (described in Table 2) generates 4 possible combination of Grid-Machine subsets to allocate tasks for a Greedy Scheduler *objectiveType*, as illustrated in Table 6. Function $f_s()$ (described in Eq. (5)) computes a preference matrix for scheduling each task on each of the generated Grid-Machine subsets, as shown in Table 7. Then, Algorithm 2 computes the schedule sequence of task allocations onto Grid-Machines, as depicted in Table 8.

**Table 6:** Grid-Machine subsets generated by $f_g()$ for *objectiveType*=$G_{CP}$.

*GMSubset*	
$$\{G_1M_1\}$$	
$$\{G_2M_1\}$$	
$$\{G_2M_2\}$$	
$$\{G_2M_1, G_2M_2\}$$

**Table 7:** Function $f_s(task, gmSubset)$ output for *objectiveType*=$G_{CP}$.

	$T_1$	$T_2$	$T_3$	$T_4$
$\{G_1M_1\}$	30.6	31.0	30.3	30.3
$\{G_2M_1\}$	30.6	31.0	30.3	30.3
$\{G_2M_2\}$	30.6	31.0	30.3	30.3
$\{G_2M_1, G_2M_2\}$	60.6	61.0	60.3	60.3

**Table 8:** Schedule sequence of tasks allocated to Grid-machines for *objectiveType*=$G_{CP}$.

Time (s)	Available tasks	freeGMs			$max(f_{s}())$	Generated schedule
		$G_1M_1$	$G_2M_1$	$G_2M_2$		
0	$T_1$	$\checkmark$	$\checkmark$	$\checkmark$	60.6	$T_1 \rightarrow \$\{G_2M_1, G_2M_2\}$
1.5	$T_2$	$\checkmark$	$\checkmark$	$\checkmark$	61.0	$T_2 \rightarrow \{G_2M_1, G_2M_2\}$
3	$T_3, T_4$	$\checkmark$	$\checkmark$	$\checkmark$	60.33	$T_3 \rightarrow \{G_2M_1, G_2M_2\}$
3	$T_4$	$\checkmark$			60.33	$T_4 \rightarrow \{G_1M_1\}$
6						Complete; $TAT = 6s$

### Objective type: greedy no fragmentation scheduler

Function $f_g()$ (described in Table 2) generates 3 possible combination of Grid-Machine subsets to allocate tasks for the Greedy Scheduler *objectiveType*, as illustrated in Table 9. Function $f_s()$ (described in Eq. (5)) computes a preference matrix for scheduling each task on each of the generated Grid-Machine subsets, as shown in Table 10. Then, Algorithm 2 computes the schedule sequence of task allocations onto Grid-Machines, as shown in Table 11.

**Table 9:** Grid-machine subsets generated by $f_g()$ for *objectiveType*=$Greedy_{NF}$.

*GMSubset*	
$$\{G_1M_1\}$$	
$$\{G_2M_1\}$$	
$$\{G_2M_2\}$$

**Table 10:** Function $f_s(task, gmSubset)$ output for *objectiveType*=$G_{NF}$.

	$T_1$	$T_2$	$T_3$	$T_4$
$\{G_1M_1\}$	60.6	61.0	60.3	60.3
$\{G_2M_1\}$	60.6	61.0	60.3	60.3
$\{G_2M_2\}$	60.6	61.0	60.3	60.3

**Table 11:** Schedule sequence of tasks allocated to grid-machines for *objectiveType*=$G_{NF}$.

Time (s)	Available tasks	freeGMs			$max(f_{s}())$	Generated schedule
		$G_1M_1$	$G_2M_1$	$G_2M_2$		
0	$T_1$	$\checkmark$	$\checkmark$	$\checkmark$	60.6	$T_1 \rightarrow \$\{G_1M_1\}$
3	$T_2$	$\checkmark$	$\checkmark$	$\checkmark$	61.0	$T_2 \rightarrow \{G_1M_1\}$
6	$T_3, T_4$	$\checkmark$	$\checkmark$	$\checkmark$	60.33	$T_3 \rightarrow \{G_1M_1\}$
6	$T_4$	$\checkmark$	$\checkmark$		60.33	$T_4 \rightarrow \{G_2M_2\}$
9		$\checkmark$	$\checkmark$	$\checkmark$		Complete; $TAT = 9s$

### Objective type: greedy execution price scheduler

Function $f_g()$ (described in Table 2) generates 4 possible combination of Grid-Machine subsets to allocate tasks for the Greedy Scheduler *objectiveType*, as illustrated in Table 12. Function $f_s()$ (described in Eq. (5)) computes a preference matrix to schedule a task on each of the generated Grid-Machine subsets, as shown in Table 13. Then, Algorithm 2 computes the schedule sequence of task allocations onto Grid-Machines, as shown in Table 14.

**Table 12:** Grid-machine subsets generated by $f_g()$ for *objectiveType*=$Greedy_{EP}$.

*GMSubset*
$$\{G_1M_1\}$$
$$\{G_2M_1\}$$
$$\{G_2M_2\}$$
$$\{G_2M_1, G_2M_2\}$$

**Table 13:** Function $f_s(task, gmSubset)$ output for *objectiveType*=$G_{EP}$.

	$T_1$	$T_2$	$T_3$	$T_4$
$\{G_1M_1\}$	66666.6	66666.6	66666.6	66666.6
$\{G_2M_1\}$	100000.0	100000.0	100000.0	100000.0
$\{G_2M_2\}$	100000.0	100000.0	100000.0	100000.0
$\{G_2M_1, G_2M_2\}$	200000.0	200000.0	200000.0	200000.0

**Table 14:** Schedule sequence of tasks allocated to Grid-Machines for *objectiveType*=$G_{EP}$.

Time (s)	Available tasks	freeGMs			$max(f_{s}())$	Generated schedule
		$G_1M_1$	$G_2M_1$	$G_2M_2$		
0	$T_1$	$\checkmark$	$\checkmark$	$\checkmark$	200000	$T_1 \rightarrow \{G_2M_1, G_2M_2\}$
1.5	$T_2$	$\checkmark$	$\checkmark$	$\checkmark$	200000	$T_2 \rightarrow \{G_2M_1, G_2M_2\}$
3	$T_3, T_4$	$\checkmark$	$\checkmark$	$\checkmark$	200000	$T_3 \rightarrow \{G_2M_1, G_2M_2\}$
3	$T_4$	$\checkmark$			66666.6	$T_4 \rightarrow \{G_1M_1\}$
6		$\checkmark$	$\checkmark$	$\checkmark$		Complete; $TAT = 6s$

## Results and discussion

### Simulation setup

The proposed multi objective task scheduling framework is simulated using GridSim. Simulation is carried out on three types of task graphs : standard task graphs, random task graphs and scientific task graphs on ubuntu operating system with AMD Ryzen 5 processor.

The framework includes five distinct task schedulers, each designed to optimize different target objectives:

1. Greedy scheduler: Prioritizes minimizing turnaround time, communication cost, and execution cost while maximizing grid utilization. 2. Greedy Communication Cost scheduler: Focused on minimizing communication cost by distributing tasks across computing resources within a single Grid. 3. Greedy Execution Cost scheduler: Aims to minimize execution cost by scheduling each task on the most suitable subset of computing resources based on their cost-to-performance ratio. 4. Greedy No Fragmentation scheduler: Aims to schedule tasks on individual computing resources, resulting in zero task fragmentation. 5. Random scheduler: Schedules tasks on a random subset of computing resources.

Table 15 explicates the notations used in the mathematical models and algorithms. Table 16 delineates the symbols representing various scheduling algorithms, while Table 17 furnishes a catalogue of scientific application graphs used in the current study.

**Table 15:** Key notation definitions.

Notation	Description
*n*	Number of tasks
*m*	Number of Grids present on the grid network
$W_{T_i}$	Length (in millions of instructions) of task $T_i$
$W_{G_j}$	Processing Capacity in MIPS (millions of instructions per second) of a single machine belonging to Grid $G_j$
*p*[1, ..., *m*]	Number of machines present on Grids $G_1,..., G_m$
$Price_{E_{G_j}}$	Price (cost) incurred per second in executing a task on any machine belonging to Grid $G_j$
$Price_{C}$	Price(cost) incurred per second in reserving the network link connecting any two different Grids on the Grid Network
$d^+(T_i)$	Out degree of Task $T_i$ on the task dependency graph i.e. the number of child tasks dependent on Task $T_i$
*GS*	Greedy scheduler - Minimize TAT and maximize Resource Utilization
*GCPS*	Greedy communication price scheduler - Minimize the communication Price (Cost)
*GEPS*	Greedy execution price scheduler - Minimize the execution Price (Cost)
*GNFS*	Greedy No-Fragmentation scheduler - Minimize TAT and maximize Resource Utilization without fragmenting any task across multiple Grid-Machines
*R*	Random scheduler

**Table 16:** Schedulers and symbols.

Scheduler name	Symbol used
Greedy scheduler	●
Greedy communication cost scheduler	⧫
Random scheduler	■
Greedy execution cost scheduler	▲
Greedy no fragmentation scheduler	★

**Table 17:** Scientific application graphs.

Scientific application workflow	Brief description
Epigenomics	Created by the USC Epigenome Center and the Pegasus Team to automate various operations in genome sequence processing.
Cybershake	Used by the Southern California Earthquake Center to characterize earthquake hazards in a region.
Gausian elimination	An algorithm for solving linear equations
LIGO	Used to generate and analyze gravitational wave forms from data collected during the coalescing of compact binary systems.
Montage	Created by NASA/IPAC to stitch together multiple input images to create custom mosaics of the sky
Cascade	User level library allowing manual pluralization of complex C++ systems such as video game engines

The proposed task scheduling algorithm is evaluated using standard, random and scientific task graphs.

### Standard task graphs

Our earlier research, as presented in^63^, demonstrated theorems for standard unit size task graphs on a homogeneous grid network for turnaround time. Similarly, in^64^, we stated theorems for grid utilization. In this article, we have formulated mathematical models for homogeneous standard-weighted task graphs on a homogeneous grid network for both fragmented and non-fragmented versions of the task graphs. These formulations are defined in Tables 18 and 19 respectively.

**Table 18:** TAT for weighted fragmented standard task graphs.

Task graph	TAT
Pipe line	$\frac{W_T}{W_{G} \times m \times M} \times n$
Star	$\frac{W_T}{W_{G} \times m \times M} + \frac{W_T \times (n - 1)}{W_{G} \times m \times M}$
Independent	$\frac{W_T \times n}{W_{G} \times m \times M}$
Binary	$\sum _{i=0}^{\ln {(n-1)} - 1} \frac{2^i \times W_T}{W_{G} \times m \times M}$
$\alpha$ ary	$\sum _{i=0}^{\ln _\alpha (n(\alpha -1)+1)-1} \frac{\alpha ^i \times W_T}{W_{G} \times m \times M}$
Fully connected	$\frac{W_T}{W_{G} \times m \times M} \times n$

**Table 19:** TAT for weighted non-fragmented standard task graphs.

Task graph	TAT
Pipe line	$\frac{W_T}{W_{G}} \times n$
Star	$\frac{W_T}{W_{G}} + \left( \frac{W_T}{W_{G}} \times \lceil \frac{n-1}{m \times M}\rceil \right)$
Independent	$\frac{n \times W_T}{W_{G} \times m \times M}$
Binary	$\le \sum _{i=0}^{\ln {(n-1)} - 1} \left( \lceil \frac{2^i}{m \times p} \rceil \times \frac{W_T}{W_{G}} \right)$
$\alpha$ ary	$\le \sum _{i=0}^{\ln _\alpha {n(\alpha -1)+1}-1} \left( \lceil \frac{\alpha ^i}{m \times M} \rceil \times \frac{W_T}{W_{G}} \right)$
Fully connected	$\frac{W_T}{W_{G} \times m \times M} \times n$

TAT obtained from the proposed algorithm is tabulated in Table 20. The result describes both theoretical and simulated results for various standard task graphs (with fragmentation and without fragmentation) for a given number of tasks, grids, and processing elements. Here each task contains a uniform number of instructions ($W_{Ti}=20000$ MI) and homogeneous processing elements ( $W_{GR}=500$ MIPS) in each grid. Computed TAT is on par with our mathematical formulations. From the results, it is evident that as number of tasks increases, TAT also increases. Similarly, The computed values of turnaround time, execution cost, communication cost, and resource utilization using proposed schedulers for pipeline, star, ternary, independent and fully connected task graphs with varying number of task nodes and a given number of grid resources are tabulated in Tables 21, 22 and 23 respectively. From these results, it has been found that the greedy scheduler successfully optimizes for the fastest turnaround time along with grid utilisation, but the trade-off is that the communication cost is high. However, the greedy communication cost scheduler with a slightly slower TAT successfully incurs the lowest communication cost. In the absence of task fragmentation, a greedy scheduler achieves optimal grid utilization while incurring zero communication costs. Additionally, it’s worth noting that the execution cost remains consistent across different task schedulers when standard graphs are processed on a homogeneous grid network. As the nature of the graphs becomes increasingly independent (such as star graphs or independent graphs), most schedulers yield similar turnaround times due to the reduced dependency constraints. The Random scheduler, inherently achieves TAT, Resource Utilization, and Communication cost in between the extremes achieved by the other schedulers. Another interesting observation is that greedy scheduler achieves maximum resource utilization and minimum turnaround time, albeit by incurring the highest communication and execution costs.

**Table 20:** Simulated/computed TAT for standard task graphs.

Standard task graph	Number of tasks (n)	TAT with fragmentation		TAT without fragmentation	
		TAT (Table 18) in seconds	Grid sim TAT in seconds	TAT (Table 19) in seconds	Grid sim TAT in seconds
Independent task graph	40	200	200.03	200	200.03
	121	605	605.1	640	640.1
	363	1820	1820.03	1840	1840.04
	1093	5465	5465.91	5480	5480.11
Star task Graph	40	200	200.04	240	240
	121	605	605.11	640	640.01
	364	1820	1820.31	1880	1880.04
	1093	5465	5465.92	5520	5520.11
$\alpha -ary$ Task graph (a=3)	40	200	200.04	320	280.01
	121	605	605.11	760	680.01
	364	1820	1820.31	2000	1880.04
	1093	5465	5465.92	5680	5560.04
Pipeline task graph	40	200	200.26	1600	1600.03
	121	605	605.8	4840	4840.1
	364	1820	1822.42	14560	14560.3
	1093	5465	5472.28	43720	43720.91
Fully connected task graph	40	200	200.26	200	200.26
	121	605	605.8	605	605.8
	364	1820	1822.42	1820	1822.42
	1093	5465	5472.28	5465	5472.28

### Random task graphs

**Figure 3:**
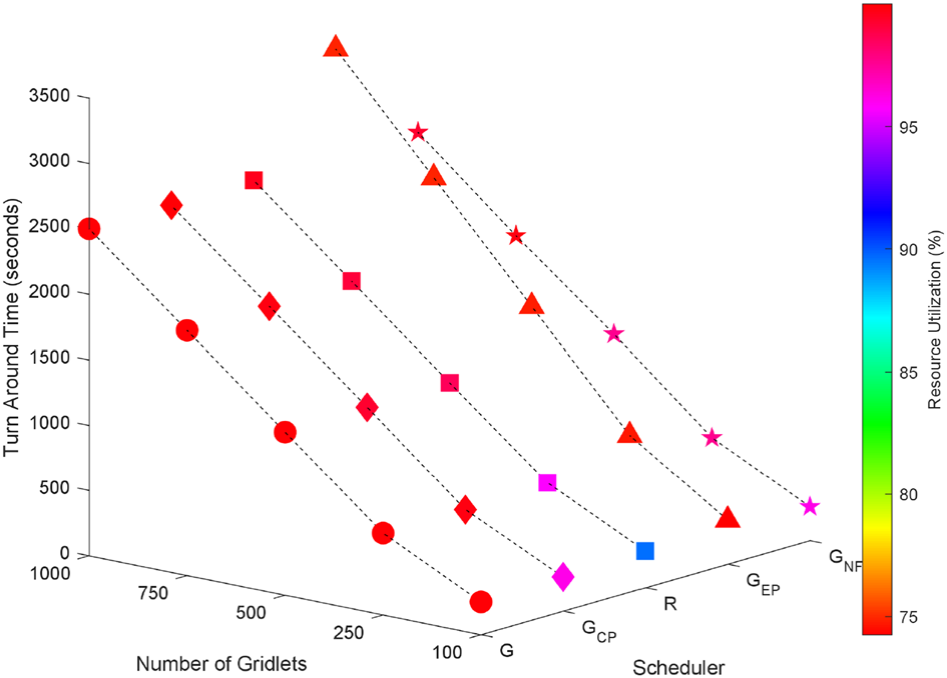
Random task graph with 0% connectivity - TAT and resource utilization.

**Figure 4:**
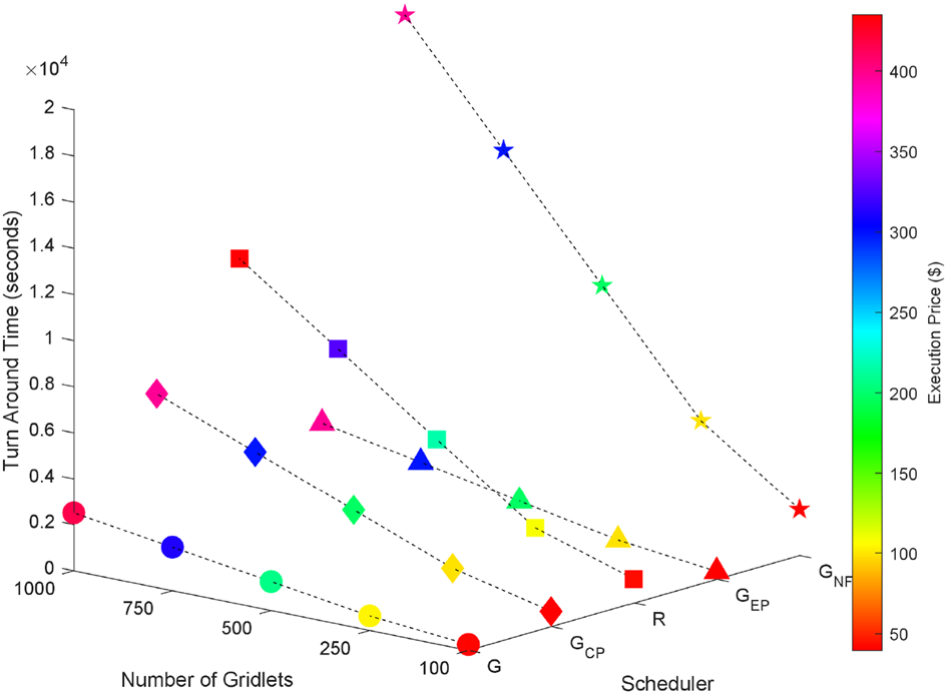
Random task graph with 100% connectivity - TAT and execution cost.

**Figure 5:**
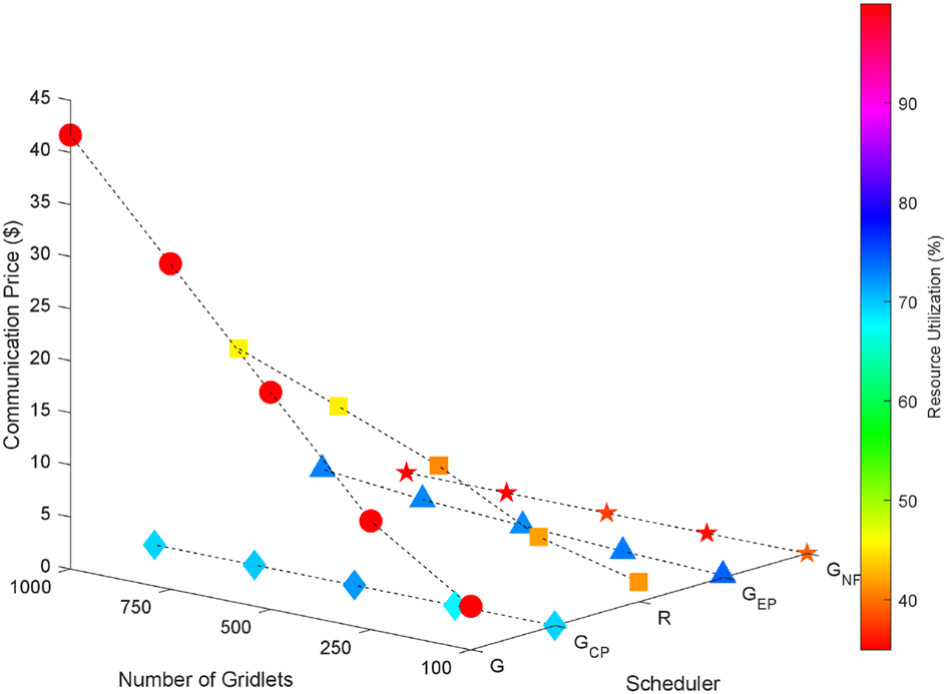
Random task graph with 25% connectivity - TAT and resource utilization.

**Figure 6:**
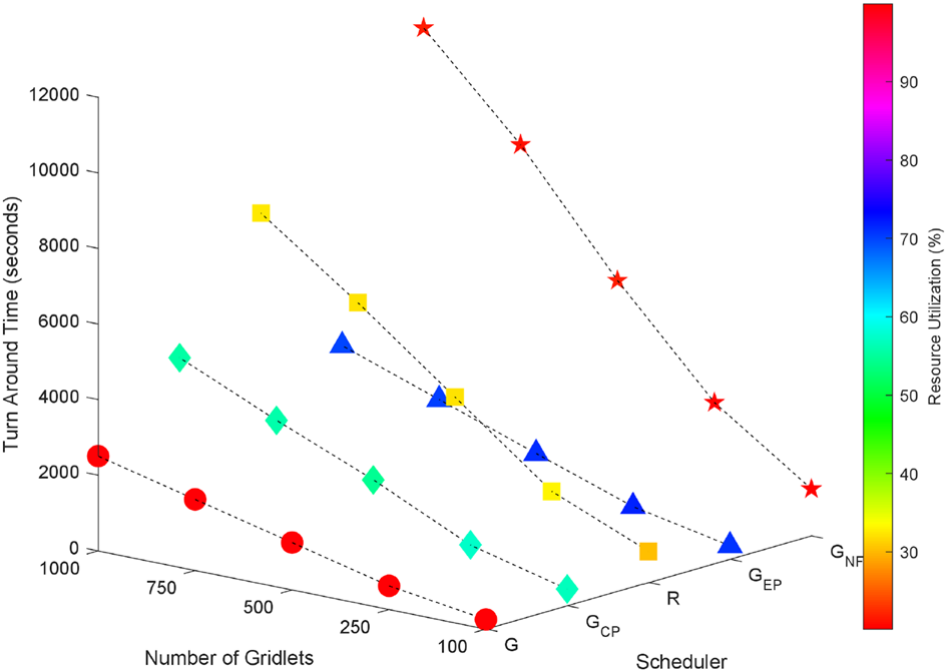
Random task graph with 50% connectivity - TAT and resource utilization.

**Figure 7:**
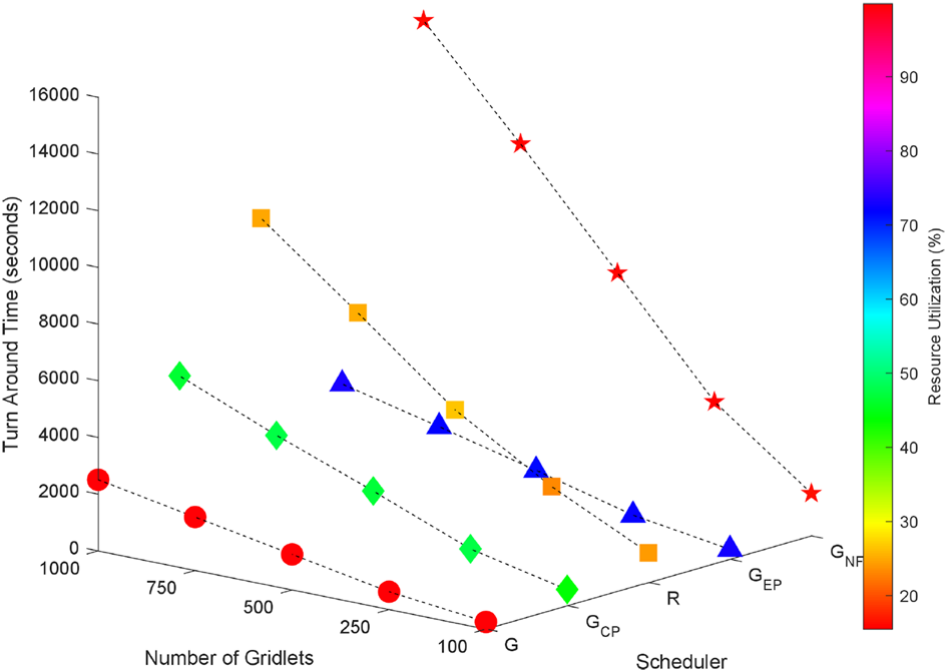
Random task graph with 75% connectivity - TAT and resource utilization.

**Figure 8:**
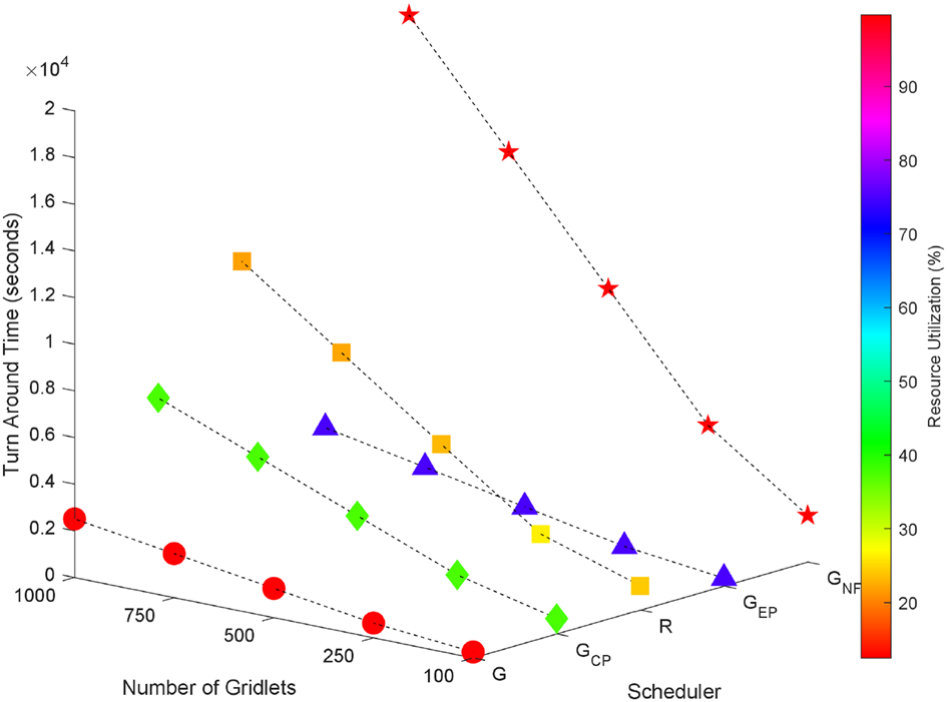
Random task graph with 100% connectivity - TAT and resource utilization.

Random task graphs with diverse levels of connectivity (0%, 25%, 50%, 75%, and 100%) is generated by using algorithm-2^64^. The outcomes of our proposed algorithm, encompassing TAT, resource utilization, execution cost, and communication cost, are depicted in Fig. 3 through Fig. 4. From these results, we can conclude that the turnaround time increases due to the increase in the number of tasks and also the increase in task dependency. This is shown in Figs. 5, 6, 7 and 8. Also when tasks are scheduled without fragmentation TAT increases as compared to tasks with fragmentation.

Resource utilization decreases when scheduling a random task graph with a higher degree of dependency without fragmentation (as seen in Figs. 5, 6, 7, and 8), in contrast to when tasks are fragmented. Additionally, it’s noteworthy that all schedulers perform optimization for turnaround time and resource utilization when there is no inter dependency among the tasks, as illustrated in Fig. 3.

**Figure 9:**
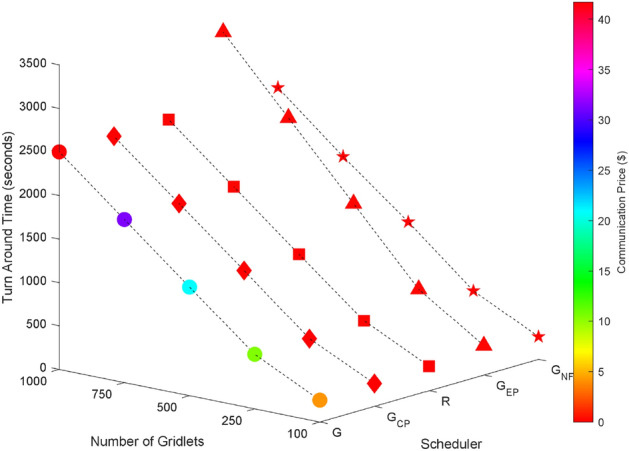
Random task graph with 0% connectivity - TAT and communication cost.

**Figure 10:**
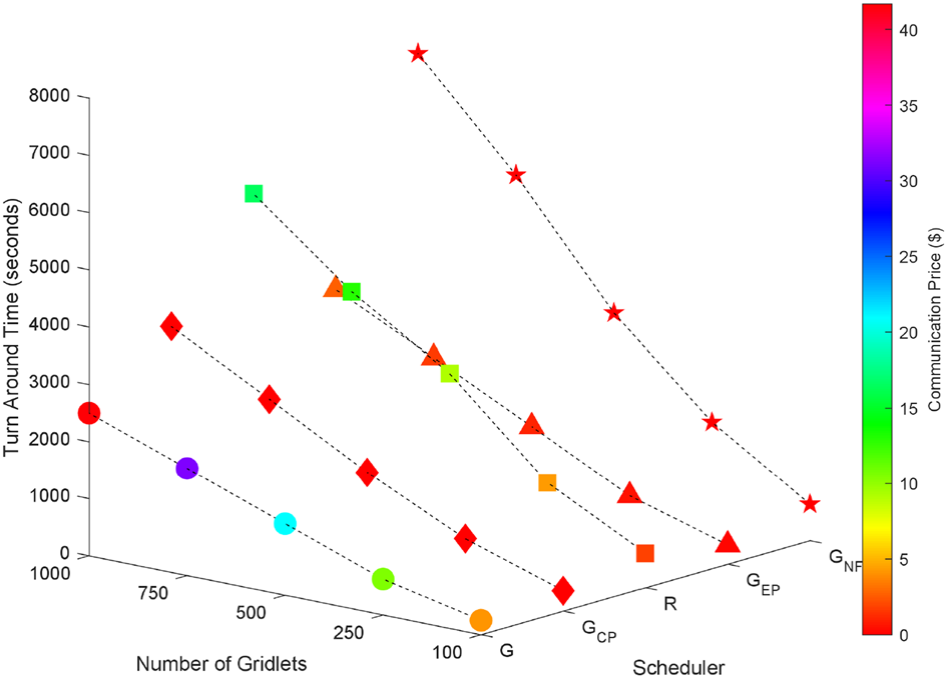
Random task graph with 25% connectivity - TAT and Commutation Cost.

**Figure 11:**
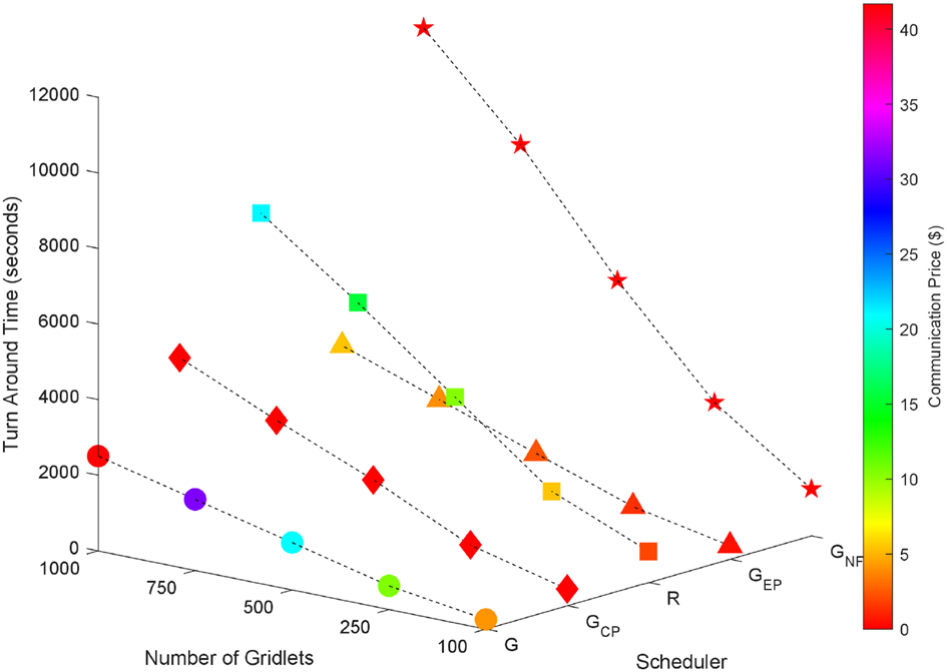
Random task graph with 50% connectivity - TAT and communication cost.

**Figure 12:**
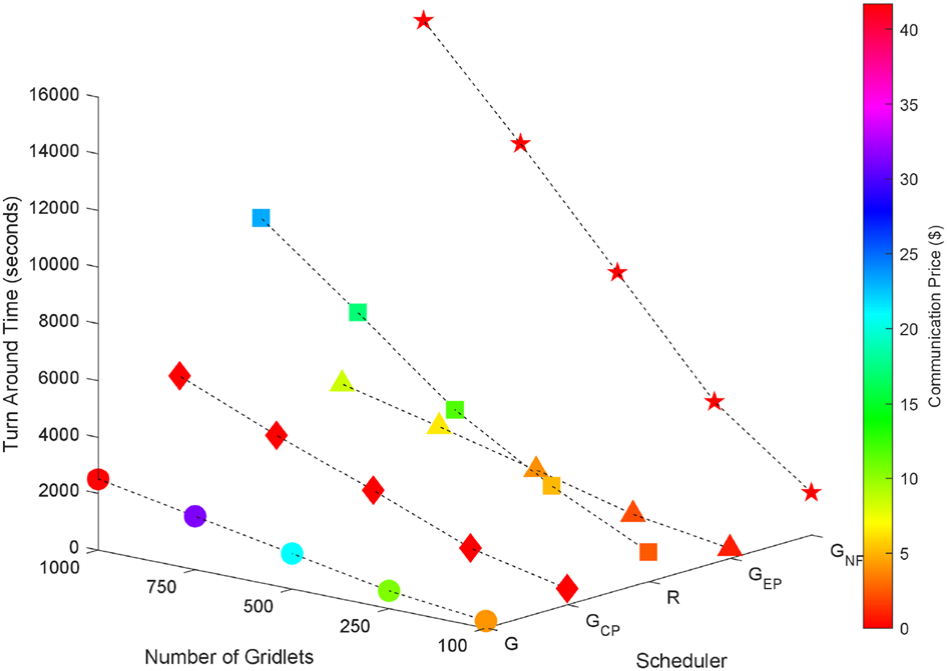
Random task graph with 75% connectivity - TAT and communication cost.

**Figure 13:**
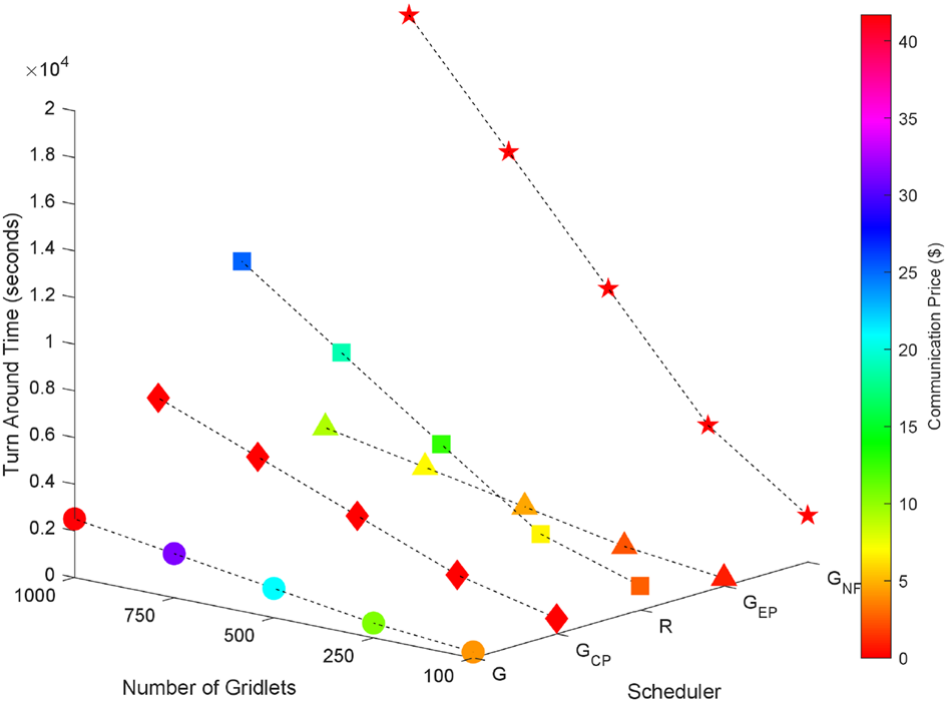
Random task graph with 100% connectivity - TAT and communication cost.

As the inter dependency between tasks within a task graph increases (with connectivity’s of 0% as shown in Fig. 9, 25% connectivity as shown in Fig. 10, 50% in Fig. 11, 75% in Fig. 12, and 100% in Fig. 13), it becomes evident that the greedy scheduler achieves the lowest Turnaround Time (TAT). However, this comes at the cost of higher communication expenses due to the fragmentation of tasks. Conversely, a greedy scheduler without task fragmentation incurs zero communication costs in all cases, effectively eliminating this expense from the scheduling process.

**Figure 14:**
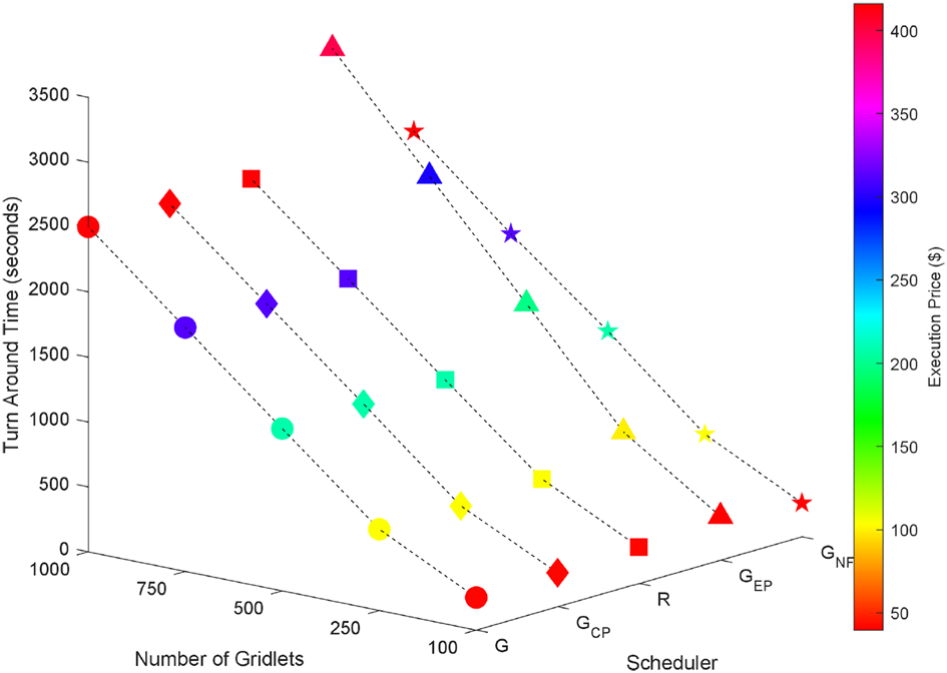
Random task graph with 0% connectivity - TAT and execution cost.

**Figure 15:**
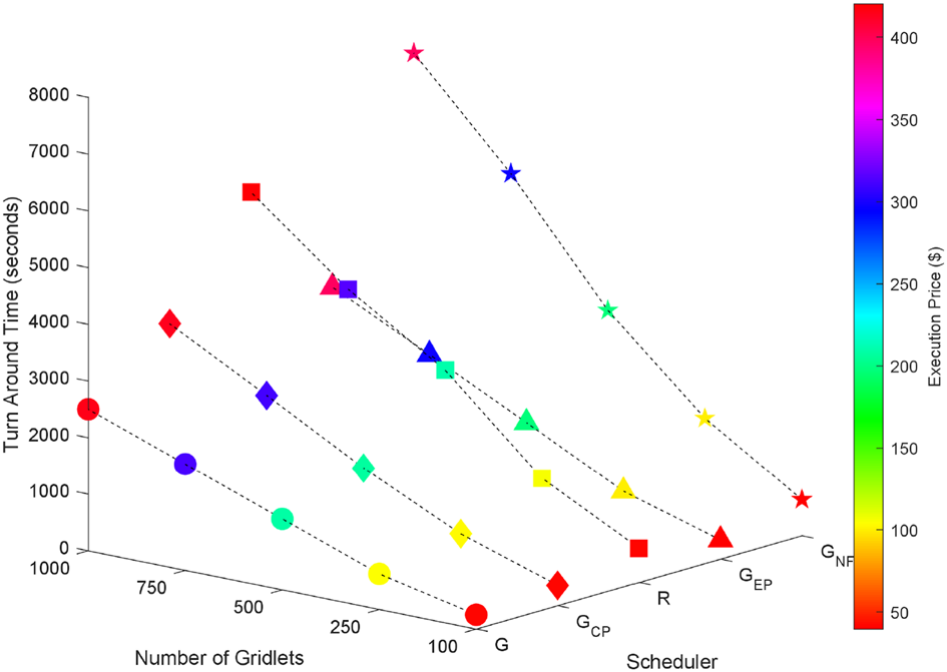
Random task graph with 25% connectivity - TAT and execution cost.

**Figure 16:**
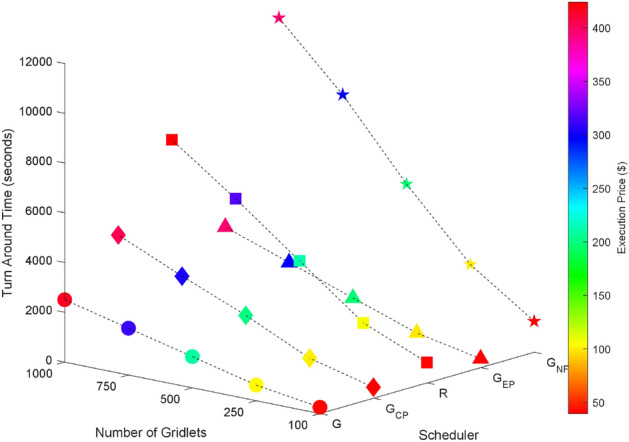
Random task graph with 50% connectivity - TAT and execution cost.

**Figure 17:**
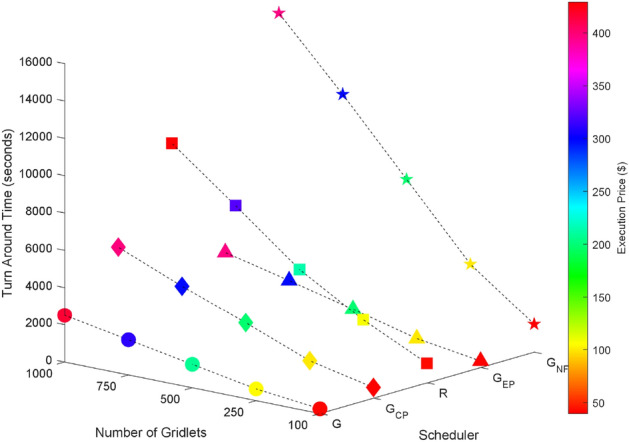
Random task graph with 75% connectivity - TAT and execution cost.

Figures 4, 14, 15, 16 and 17 shows that the greedy execution cost scheduler incurs the least execution cost. The computed values of turnaround time, execution cost, communication cost, and resource utilization using proposed schedulers for random task graphs with 25%, 50%, 75% and 100% dependency with varying number of task nodes and a given number of grid resources are tabulated in Tables  24, 25 and 26 respectively.

**Table 21:** Performance of standard task graphs on AWS EC2 Type-1, Type- 2, 2 Grids and 11 CPUs.

Number of gridlets (n)	Scheduler	Standard task graphs							
		Pipeline task graph				Star task graph			
		Turn around time (seconds)	Resource utilization (%)	Execution cost ($)	Communication cost ($)	Turn around time (seconds)	Resource utilization (%)	Execution cost ($)	Communication cost ($)
40	Greedy (with fragmentation) scheduler	89.24	99.61	16.54	88.88	84.47	105.23	15.71	64.44
	Greedy communication cost scheduler	114.51	77.63	15.87	0	92.87	95.71	16.55	0
	Random scheduler	380.68	23.35	17.66	88.29	99.22	89.59	16.53	12.21
	Greedy execution cost scheduler	800.03	11.11	15.87	0	120	74.07	16.48	0
	Greedy (without-fragmentation) scheduler	800.03	11.11	15.87	0	120	74.07	16.48	0
121	Greedy (with fragmentation) scheduler	269.96	99.6	50.04	268.86	234.48	114.67	43.25	64.44
	Greedy communication cost scheduler	346.4	77.62	48.01	0	272.88	98.54	50.04	0
	Random scheduler	1335.94	20.13	53.73	272.51	294.01	91.46	50.16	44
	Greedy execution cost scheduler	2420.1	11.11	48.02	0	300.01	89.63	50.13	0
	Greedy (without-fragmentation) scheduler	2420.1	11.11	48.02	0	300.01	89.63	50.13	0
364	Greedy (with fragmentation) scheduler	812.14	99.6	150.53	808.81	675.89	119.68	125.69	64.44
	Greedy communication cost scheduler	1042.07	77.62	144.44	0	812.93	99.5	150.52	0
	Random scheduler	3683.09	21.96	161.88	892.11	844.34	95.8	150.68	18.33
	Greedy execution cost scheduler	7280.3	11.11	144.44	0	840.02	96.29	150.48	0
	Greedy(without-Fragmentation) scheduler	7280.3	11.11	144.44	0	840.02	96.29	150.48	0
1039	Greedy (with fragmentation) scheduler	2318.17	99.6	429.67	2308.66	1904.62	121.23	354.34	64.44
	Greedy communication cost scheduler	2974.48	77.62	412.28	0	2313.05	99.82	429.63	0
	Random scheduler	11322.52	20.39	462.72	2492.34	2350.67	98.22	429.77	71.66
	Greedy execution cost scheduler	20780.86	11.11	412.3	0	2340.05	98.67	429.79	0
	Greedy (without-Fragmentation) scheduler	20780.86	11.11	412.3	0	2340.05	98.67	429.79	0

**Table 22:** Performance of standard task graphs on AWS EC2 Type-1, Type- 2, 2 Grids and 11 CPUs.

Number of gridlets (n)	Scheduler	Standard task graphs							
		Independent task graph				Ternary task graph			
		Turn around time (seconds)	Resource utilization (%)	Execution cost ($)	Communication cost ($)	Turn around time (seconds)	Resource utilization (%)	Execution cost ($)	Communication cost ($)
40	Greedy (with fragmentation) scheduler	84.45	105.25	15.61	62.22	84.47	105.23	15.71	64.44
	Greedy communication cost scheduler	90.01	98.76	16.55	0	92.87	95.71	16.55	0
	Random scheduler	109	81.55	16.67	25.67	106.67	83.33	16.58	43.11
	Greedy execution cost scheduler	100	88.89	16.48	0	140	63.49	16.48	0
	Greedy (without-fragmentation) scheduler	100	88.89	16.48	0	140	63.49	16.48	0
121	Greedy (with fragmentation) scheduler	232.25	115.77	43.15	62.22	234.48	114.67	43.25	64.44
	Greedy communication cost scheduler	270.02	99.58	50.04	0	272.88	98.54	50.04	0
	Random scheduler	284	94.68	50.08	4	294.34	91.35	50.09	15.08
	Greedy execution cost scheduler	280.01	96.03	50.13	0	320	84.03	49.98	0
	Greedy (without-fragmentation) scheduler	280.01	96.03	50.13	0	320	84.03	49.98	0
364	Greedy (with fragmentation) scheduler	682.29	118.56	125.75	62.22	675.89	119.68	125.69	64.44
	Greedy communication cost scheduler	810.06	99.86	150.52	0	812.93	99.5	150.52	0
	Random scheduler	821	98.52	150.54	21.33	835.01	96.87	150.58	5
	Greedy execution cost scheduler	820.02	98.64	150.48	0	860	94.06	150.48	0
	Greedy (without-fragmentation) scheduler	820.02	98.64	150.48	0	860	94.06	150.48	0
1039	Greedy (with fragmentation) scheduler	1904.46	121.24	354.24	62.22	1904.62	121.23	354.34	64.44
	Greedy communication cost scheduler	2310.19	99.94	429.63	0	2313.05	99.82	429.63	0
	Random scheduler	2332.78	98.98	429.74	17.78	2368.68	97.48	429.8	15.67
	Greedy execution cost scheduler	2320.05	99.52	429.79	0	2360	97.83	429.64	0
	Greedy (without-fragmentation) scheduler	2320.05	99.52	429.79	0	2360	97.83	429.64	0

**Table 23:** Perform acne of fully connected task graphs on AWS EC2 Type-1, Type- 2, 2 Grids and 11 CPUs.

Number of gridlets (n)	Scheduler	Standard task graphs			
		Fully connected task graph			
		Turn around time (seconds)	Resource utilization (%)	Execution cost ($)	Communication cost ($)
40	Greedy (with fragmentation) scheduler)	89.24	99.61	16.54	88.88
	Greedy communication cost scheduler	114.51	77.63	15.87	0
	Random scheduler	341.24	26.05	17.59	98.41
	Greedy execution cost scheduler	800.03	11.11	15.87	0
	Greedy (without-fragmentation) scheduler	800.03	11.11	15.87	0
121	Greedy (with fragmentation) scheduler	269.96	99.6	50.04	268.86
	Greedy communication cost scheduler	346.4	77.62	48.01	0
	Random scheduler	1332.88	20.17	53.97	259.55
	Greedy execution cost scheduler	2420.1	11.11	48.02	0
	Greedy (without-fragmentation) scheduler	2420.1	11.11	48.02	0
364	Greedy (with fragmentation) scheduler	812.14	99.6	150.53	808.81
	Greedy communication cost scheduler	1042.07	77.62	144.44	0
	Random scheduler	3872.95	20.89	161.64	874.12
	Greedy execution cost scheduler	7280.3	11.11	144.44	0
	Greedy (without-fragmentation) scheduler	7280.3	11.11	144.44	0
1039	Greedy (with fragmentation) scheduler	2318.17	99.6	429.67	2308.66
	Greedy communication cost scheduler	2974.48	77.62	412.28	0
	Random scheduler	11190.07	20.63	461.31	2516.51
	Greedy execution cost scheduler	20780.86	11.11	412.3	0
	Greedy (without-fragmentation) scheduler	20780.86	11.11	412.3	0

**Table 24:** Performance of scheduling algorithms on random task graphs on AWS EC2 Type-1, Type- 2, 2 Grids and 11 CPUs.

Number of gridlets (n)	Scheduler	Task graph							
		A task graph with 0% connectivity				A task graph with 25% connectivity			
		TAT (seconds)	Resource utilization (%)	Execution cost ($)	Communication cost ($)	TAT (seconds)	Resource utilization (%)	Execution cost ($)	Communication cost ($)
100	Greedy (with fragmentation) scheduler	193.97	99.56	36.01	62.22	224.56	98.96	38.51	84.44
	Greedy communication cost scheduler	222.87	98	41.33	0	248.6	89.39	41.86	0
	Random scheduler	246	90.33	41.37	6.67	550.5	40.47	41.86	194.24
	Greedy execution cost scheduler	240	92.59	41.42	0	680.03	32.68	39.68	0
	Greedy (without-fragmentation) scheduler	240	92.59	41.42	0	779.01	28.53	39.66	0
300	Greedy (with fragmentation) scheduler	562.28	118.57	103.95	62.22	651.03	102.4	115.09	217.76
	Greedy communication cost scheduler	670.05	99.49	124.08	0	781.51	85.31	124.63	0
	Random scheduler	690	96.62	124.14	23.33	1794.81	37.14	126.11	493.82
	Greedy Execution cost scheduler	680.01	98.04	124.17	0	2037.06	32.73	118.98	0
	Greedy (without-Fragmentation) scheduler	680.01	98.04	124.17	0	2113.01	31.55	118.9	0
500	Greedy (with fragmentation) scheduler	922.55	120.44	171.64	62.22	1133.6	98.02	196.05	275.53
	Greedy communication cost scheduler	1111.46	99.97	206.74	0	1224.37	90.75	207.21	0
	Random scheduler	1119	99.29	206.76	16.67	2860.33	38.85	210.25	902.54
	Greedy execution cost scheduler	1120.02	99.2	206.86	0	3652.14	30.42	198.25	0
	Greedy (without-fragmentation) scheduler	1120.02	99.2	206.86	0	3329.08	33.38	198.19	0

**Table 25:** Performance of scheduling algorithms on random task graphs on AWS EC2 Type-1, Type- 2, 2 Grids and 11 CPUs.

Number of gridlets (n)	Scheduler	Task graph							
		A task graph with 50% connectivity				A task graph with 5% connectivity			
		TAT (seconds)	Resource utilization (%)	Execution cost ($)	Communication cost ($)	TAT (seconds)	Resource utilization (%)	Execution cost ($)	Communication cost ($)
100	Greedy (with fragmentation) scheduler	242.96	91.46	41	159.98	226.63	98.06	41.35	211.09
	Greedy communication cost scheduler	320.13	69.42	41.61	0	337.34	65.88	41.33	0
	Random scheduler	854.68	26	42.78	189.18	874.23	25.42	43.3	193.28
	Greedy execution cost scheduler	1039.06	21.39	39.66	0	1600.08	13.89	39.68	0
	Greedy(without-fragmentation) scheduler	1099.05	20.22	39.66	0	1660.08	13.39	39.68	0
300	Greedy (with fragmentation) scheduler	747.19	89.22	121.54	453.29	708.43	94.1	123.34	597.72
	Greedy communication cost scheduler	946.2	70.46	124.72	0	966.73	68.96	122.79	0
	Random scheduler	2574.16	25.9	129	638.88	2951.95	22.58	130.79	633.81
	Greedy execution cost scheduler	3491.15	19.1	118.87	0	4720.23	14.12	119.05	0
	Greedy (without-fragmentation) scheduler	3453.12	19.31	118.91	0	4800.23	13.89	119.05	0
500	Greedy (with fragmentation) scheduler	1240.61	89.56	203.32	759.92	1158.96	95.87	205.88	1026.56
	Greedy communication cost scheduler	1536.23	72.33	207.32	0	1634.52	67.98	204.31	0
	Random scheduler	3821.02	29.08	214.51	1003.02	5088.05	21.84	218.14	1091.33
	Greedy execution cost scheduler	5820.31	19.09	198.41	0	7960.38	13.96	198.41	0
	Greedy(without-Fragmentation) scheduler	5872.29	18.92	198.25	0	7920.38	14.03	198.41	0

**Table 26:** Performance of scheduling algorithms on random task graphs on AWS EC2 Type-1, Type- 2, 2 Grids and 11 CPUs.

Number of gridlets (n)	Scheduler	A task graph with 100% connectivity			
		TAT (seconds)	Resource utilization (%)	Execution cost ($)	Communication cost ($)
100	Greedy (with fragmentation) scheduler	223.11	99.6	41.35	222.2
	Greedy communication cost scheduler	286.28	77.62	39.68	0
	Random scheduler	1098	20.24	44.44	243.75
	Greedy execution cost scheduler	2000.08	11.11	39.68	0
	Greedy (without-Fragmentation) scheduler	2000.08	11.11	39.68	0
300	Greedy (with fragmentation) scheduler	669.34	99.6	124.06	666.6
	Greedy communication cost scheduler	858.84	77.62	119.04	0
	Random scheduler	3232.57	20.62	133.75	728.72
	Greedy execution cost scheduler	6000.25	11.11	119.05	0
	Greedy (without-fragmentation) scheduler	6000.25	11.11	119.05	0
500	Greedy (with fragmentation) scheduler	1115.57	99.6	206.77	1111
	Greedy communication cost scheduler	1431.41	77.62	198.4	0
	Random scheduler	5536.25	20.07	222.33	1176.6
	Greedy execution cost scheduler	10000.42	11.11	198.41	0
	Greedy (without-fragmentation) scheduler	10000.42	11.11	198.41	0

### Scientific task graphs

**Figure 18:**
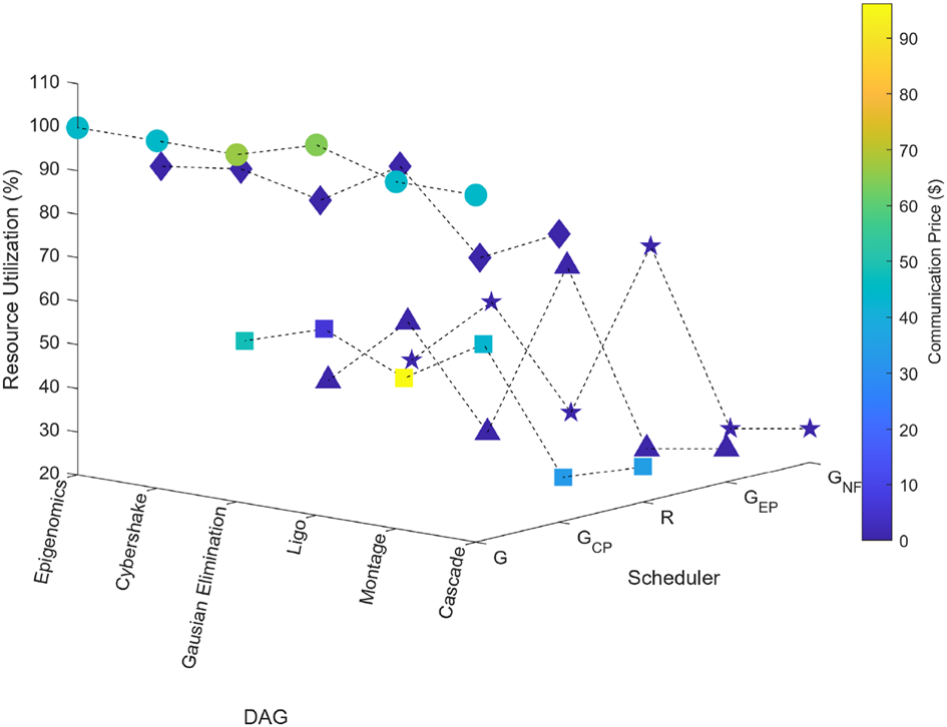
Scientific task graphs with resource utilization and communication cost.

**Figure 19:**
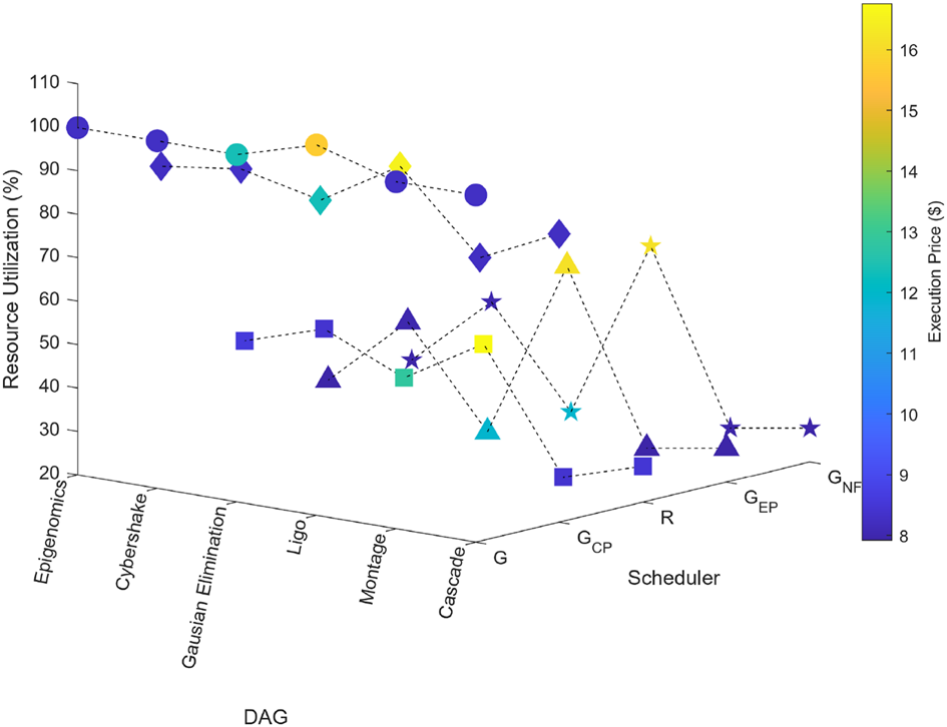
Scientific task graphs with resource utilization and execution cost.

**Figure 20:**
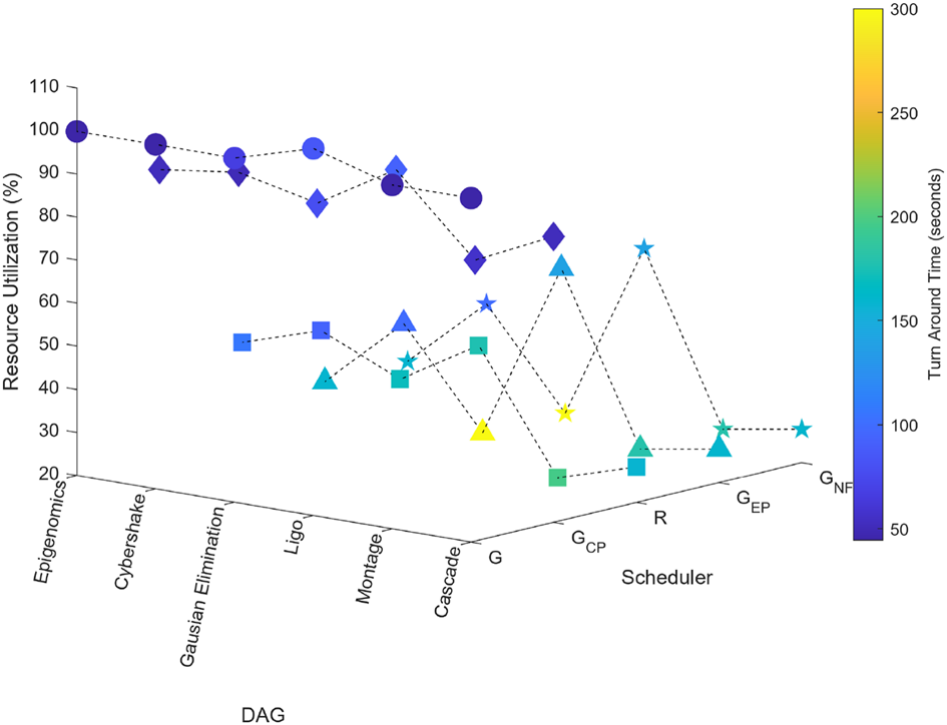
Scientific task graphs with resource utilization and TAT.

**Figure 21:**
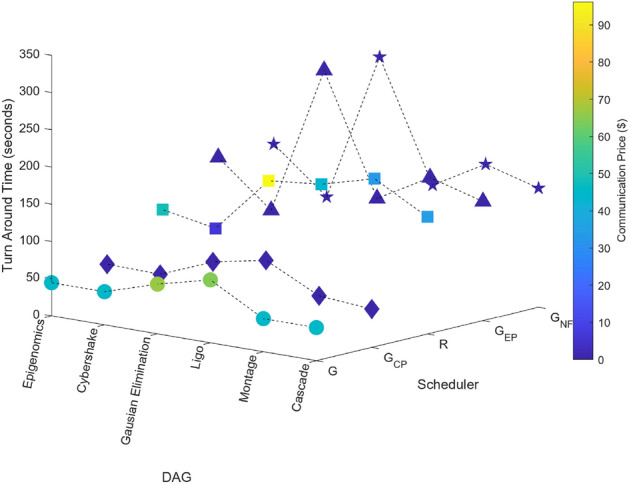
Scientific task graphs with resource utilization and communication cost.

**Figure 22:**
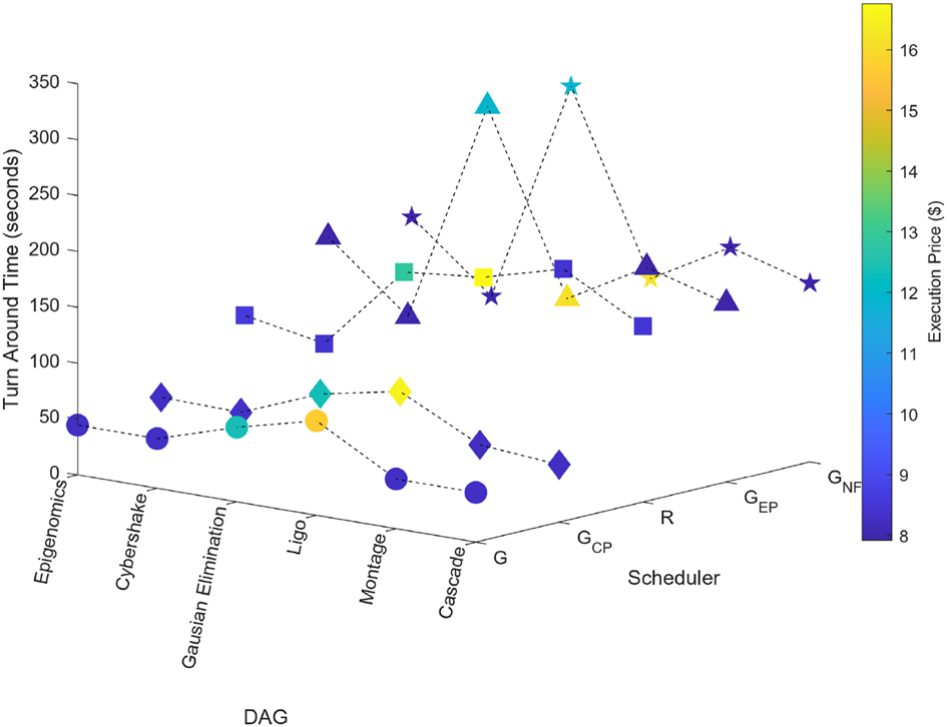
Scientific task graphs with TAT and execution cost.

The performance of the proposed algorithm is also evaluated by using scientific graphs such as Montage, CyberShake, LIGO etc. Here all workflows are generated by Pegasus Workflow Generator^65^. Results shown in Figs. 18, 19, 20, 21 and 22 demonstrates the performance of proposed algorithm.

Our observation reveals that across all application task graphs, the greedy scheduler consistently generates schedules with the most optimal TAT and resource utilization. However, it’s important to note that this optimization is achieved at the expense of incurring the highest communication and execution costs compared to the schedules generated by the other schedulers.

A consistent trend that emerges across all schedulers is the inverse relationship between resource utilization and the extent of parallel execution of tasks, which is dictated by the inter-dependency constraints among tasks. For example, in the case of the Gaussian Elimination and Montage scientific application graphs, where tasks exhibit a high degree of inter-dependency, the scheduling sequences result in the lowest resource utilization. This highlights the influence of task inter-dependency on resource allocation and utilization in the scheduling process.

Similarly, computed values of turnaround time, execution cost, communication cost, and resource utilization using proposed schedulers for different scientific task graphs is tabulated in Table 27.

**Table 27:** Performance of scheduling algorithms on scientific task graphs on AWS EC2 Type-1, Type- 2, 2 Grids and 11 CPUs.

Scientific task graph	Scheduler	TAT (seconds)	Resource utilization (%)	Execution cost ($)	Communication cost ($)
Cascade	Greedy (with fragmentation) scheduler	44.49	99.9	8.27	44.44
	Greedy communication cost schedule	51.46	86.37	8.24	0
	Random scheduler	157.33	28.25	8.49	34.68
	Greedy execution cost schedule	160	27.78	7.94	0
	Greedy (without-fragmentation) scheduler	160	27.78	7.94	0
Montage	Greedy (with fragmentation) scheduler	44.51	99.84	8.27	44.44
	Greedy Communication cost schedule	57.18	77.73	8.24	0
	Random scheduler	196.53	22.61	8.43	33.37
	Greedy Execution cost schedule	180.01	24.69	7.94	0
	Greedy (without-fragmentation) scheduler	180.01	24.69	7.94	0
Ligo	Greedy (with fragmentation) scheduler	84.47	105.24	15.71	64.44
	Greedy communication cost schedule	92.87	95.71	16.55	0
	Random scheduler	177.12	50.19	16.76	43
	Greedy execution cost schedule	140.01	63.49	16.17	0
	Greedy (without-fragmentation) scheduler	140.01	63.49	16.17	0
Gausian elimination	Greedy (with fragmentation)scheduler	66.73	99.9	12.41	66.66
	Greedy communication cost scheduler	78.62	84.79	12.36	0
	Random scheduler	169.69	39.29	12.85	96.21
	Greedy execution cost schedule	300.01	22.22	11.9	0
	Greedy (without-Fragmentation) scheduler	300.01	22.22	11.9	0
Cybershake	Greedy (with fragmentation) scheduler	44.46	99.97	8.27	44.44
	Greedy communication cost scheduler	50	88.88	8.31	0
	Random scheduler	93.67	47.45	8.42	6.67
	Greedy execution cost scheduler	100	44.44	8.01	0
	Greedy (without-fragmentation) scheduler	100	44.44	8.01	0
Epigenomics	Greedy (with fragmentation) scheduler	44.49	99.9	8.27	44.44
	Greedy communication cost scheduler	51.45	86.38	8.24	0
	Random scheduler	106.8	41.61	8.59	48.44
	Greedy execution cost scheduler	159	27.95	7.92	0
	Greedy (without-Fragmentation) scheduler	159	27.95	7.92	0

**Table 28:** TOPSIS ranking of scheduling algorithms on standard task graphs.

Standard task graph	Number of gridlets	Weightage type	TOPSIS solution rank	Scheduler
Fully connected, pipeline, ternary, star and independent	40, 121, 364 and 1039	1.TAT > RU > EP > CP 2.TAT > RU > CP > EP 3. TAT > EP > RU > CP 4. TAT > EP > CP > RU	1	Greedy (with fragmentation) scheduler
			2	Greedy Communication cost scheduler
			3	Greedy Execution cost scheduler
			4	Greedy (without-Fragmentation) scheduler
		5. TAT > CP > RU > EP 6. TAT > CP > EP > RU	1	Greedy Communication cost scheduler
			2	Greedy Execution cost scheduler
			3	Greedy (without-Fragmentation) scheduler
			4	Greedy (with fragmentation) scheduler
		7. RU > TAT > EP > CP 8. RU > TAT > CP > EP 9. RU > EP > TAT > CP 10. RU > EP > CP > TAT	1	Greedy (with fragmentation) scheduler
			2	Greedy Communication cost scheduler
			3	Greedy Execution cost scheduler
			4	Greedy (without-Fragmentation) scheduler
		11. RU > CP > TAT > EP 12. RU > CP > EP > TAT	1	Greedy Communication cost scheduler
			2	Greedy Execution cost scheduler
			3	Greedy (without-Fragmentation) scheduler
			4	Greedy (with fragmentation) scheduler

**Table 29:** TOPSIS ranking of scheduling algorithms on random task graphs.

Number of gridlets	Connectivity percentage	Weightage type	TOPSIS scheduler ranking
100, 250, 500,750 and 1000	25%, 50%, 75% and 100%	1. TAT >RU >EC >CC 2. TAT >RU >CC >EC 3. TAT >EC >RU >CC 4. TAT >EC >CC >RU 5. TAT >CC >RU >EC 6. TAT >CC >EC >RU 7. RU >TAT >EC >CP 8. RU >TAT >CC >EP 9. RU >EC >TAT >CP 10. RU >EC >CC >TAT 11. RU >CC >TAT >EP 12. RU >CC >EC >TAT	1. Greedy (with fragmentation) scheduler 2. Greedy Execution Cost scheduler 3. Greedy Communication cost scheduler 4. Greedy (without-Fragmentation) scheduler
		13.CC >TAT >RU >EC 14.CC >TAT >EC >RU 15.CC >RU >TAT >EC 16.CC >RU >EC >TAT 17.CC >EC >TAT >RU 18.CC >EC >RU >TAT	1. Greedy Communication cost scheduler 2. Greedy (without-Fragmentation) scheduler 3. Greedy Execution cost scheduler 4. Greedy (with fragmentation) scheduler
		19.EC >TAT >RU >CC 20. EC >TAT >CC >RU	1. Greedy Execution cost scheduler 2. Greedy (with fragmentation) scheduler 3. Greedy Communication cost scheduler 4. Greedy (without-Fragmentation) scheduler

**Table 30:** TOPSIS ranking of scheduling algorithms on scientific task graphs.

Scientific task graph	Number of gridlets	Weightage type	TOPSIS solution rank	Scheduler
Cascade, montage, ligo, cybershake, epigenomics and Gaussian elimination	40, 121, 364 and 1039	1.TAT > RU > EC > CC 2.TAT > RU > CC > Ec 3. TAT > EC > RU > CC 4. TAT > EC > CC > RU	1	Greedy (with fragmentation) scheduler
			2	Greedy Communication cost scheduler
			3	Greedy Execution cost scheduler
			4	Greedy (without-Fragmentation) scheduler
		5. TAT > CC > RU > EC 6. TAT > CC > EC > RU	1	Greedy Communication cost scheduler
			2	Greedy Execution cost scheduler
			3	Greedy (without-Fragmentation) scheduler
			4	Greedy (with fragmentation) scheduler
		7. RU > TAT > EC > CC 8. RU > TAT > CC > EC 9. RU > EC > TAT > CC 10. RU > EC > CC > TAT	1	Greedy (with fragmentation) scheduler
			2	Greedy Communication cost scheduler
			3	Greedy Execution cost scheduler
			4	Greedy (without-Fragmentation) scheduler
		11. RU > CC > TAT > EC 12. RU > CC > EC > TAT	1	Greedy Communication cost scheduler
			2	Greedy Execution cost scheduler
			3	Greedy (without-Fragmentation) scheduler
			4	Greedy (with fragmentation) scheduler

## Formulation of the multi-objective-decision-making problem

### The generic multi-attribute-decision-making (MADM) problem

Scheduling tasks in a grid network can be conceptualized as a MADM problem. In the context of MADM, the goal is to assess and prioritize various alternative solutions denoted as $A_i(i = 1, 2, 3, \dots , I)$, taking into account specific criteria. These criteria, represented as $C_j(j = 1, 2, 3, \dots , J)$, encapsulate the factors that play a role in influencing the ranking of the alternative solutions within the set $A_i$.

Each alternative solution, denoted as $A_i$, undergoes an evaluation against each individual criterion, represented by $C_j$. This evaluation process produces a performance rating matrix $X = (x_{ij})_{(I \times J)}$.

$$\begin{aligned} X = \begin{array}{*{20}c} A_1 \\ A_2 \\ \dots \\ A_I \\ \end{array} \mathop {\left( {\begin{array}{*{20}l} x_{11}&{} x_{12}&{} \dots &{} x_{1J}\\ x_{21}&{} x_{22}&{} \dots &{} x_{2J}\\ \dots &{} \dots &{} \dots &{} \dots \\ x_{I1}&{}x_{I2}&{} \dots &{} x_{IJ}\\ \end{array} } \right) } \limits ^{{\begin{array}{*{20}l} &{} C_1 &{} C_2 &{} \dots &{} C_j \\ \end{array} }} \end{aligned}$$

The user is tasked with specifying a set of weights, denoted as $W = w_j(j = 1, 2, \dots , J)$, which serve as indicators of the user’s individual preferences for each criterion, $C_j$.

### Modeling task scheduling as an MADM problem

We model task scheduling problem as an MADM problem by:

1. Considering the schedule sequence output by each scheduler as the set of alternative solutions i.e. $A = \{ a | a \subset \{GS, GCCS, GECS, GNFS\} \}$.
2. Considering the performance metrics of a schedule sequence as the set of criteria i.e. $C = \{c | c \subset \{TAT, RU, CC, EC\}\}$.
3. Computing the performance rating of each scheduler (*GS*, *GCCS*, *GECS*, *GNFS*) against every criteria (*TAT*, *RU*, *CC*, *EC*).
  i.e.
  $$\begin{aligned} X = \begin{array}{*{20}c} GS \\ GCCS \\ GECS \\ GNFS \\ \end{array} \mathop {\left( {\begin{array}{*{20}l} y_{11} &{} x_{12}&{} x_{13}&{} x_{14}\\ x_{21}&{} x_{22}&{} x_{23}&{} x_{24}\\ x_{31}&{} x_{32}&{} x_{33}&{} x_{34}\\ x_{41} &{} x_{42} &{} x_{43} &{} x_{44} \\ \end{array} } \right) } \limits ^{{\begin{array}{*{20}l} &{} TAT &{} RU &{} CC &{} EC \\ \end{array} }} \end{aligned}$$
4. Collecting a user’s preferences for each criterion involves ranking these criteria in descending order of importance. Weights are then allocated using a Geometric Progression, with greater weights being assigned to criteria ranked higher in importance by the user.

### Solving the MADM problem

The task scheduling MADM problem is addressed using a well-regarded technique within the MADM field known as TOPSIS. TOPSIS operates on the principle that the optimal solution is the one closest to the positive-ideal solution while simultaneously being the farthest from the negative-ideal solution. Alternatives are ranked by computing an overall index based on their proximity to these ideal solutions.

The TOPSIS method comprises a series of steps, as follows:

1. Normalize the performance rating matrix.
  i.e. $$y_{ij} = \frac{x_{ij}}{\sqrt{ \sum _{i=1}^{I} x_{ij}^2 }}$$
  $$Y = \begin{bmatrix} y_{11} &{} y_{12} &{} \dots &{} y_{1J} \\ y_{21} &{} y_{22} &{} \dots &{} y_{2J} \\ \dots &{} \dots &{} \dots &{} \dots \\ y_{I1} &{} y_{I2} &{} \dots &{} y_{IJ} \end{bmatrix}$$
2. Determine the weighted, normalized performance rating matrix.
  i.e.
  $$V = \begin{bmatrix} y_{11} &{} v_{12} &{} \dots &{} v_{1J} \\ v_{21} &{} v_{22} &{} \dots &{} v_{2J} \\ \dots &{} \dots &{} \dots &{} \dots \\ v_{I1} &{} v_{I2} &{} \dots &{} v_{IJ} \end{bmatrix}$$
  Where $$v_{ij} = W_j * y_{ij}; (i = 1, 2, \dots , I; j = 1, 2, \dots , J)$$
3. Compute the positive and negative ideal solutions, $A^+$ and $A^-$, respectively.
  $$A^+ = [v_1^+, v_2^+, \dots , v_J^+]$$ $$A^+ = [v_1^-, v_2^-, \dots , v_J^-]$$
  where,
  $$v_j^+ = {\left\{ \begin{array}{ll} max_{i=1}^{I}(v_{ij})\,\,\,\, \text {if }\,\,\,\, j \text { is a benefit attribute}, min_{i=1}^{I}(v_{ij})\,\, \text {if }\,\,\,\, j\,\,\,\, \text { is a cost attribute} \end{array}\right. }$$
  $$v_j^- = {\left\{ \begin{array}{ll} min_{i=1}^{I}(v_{ij})\,\,\,\, \text {if }\,\, j \,\,\,\,\text { is a benefit attribute}, max_{i=1}^{I}(v_{ij})\,\, \text {if } \,\,\,\,j\,\,\,\, \text { is a cost attribute} \end{array}\right. }$$
4. Calculate the Euclidean distance from the positive and negative ideal solutions.
  $$S_i^+ = \sqrt{ \sum _{j=1}^j (v_{ij} - v_{j}^+)^2 }$$
  $$S_i^- = \sqrt{ \sum _{j=1}^j (v_{ij} - v_{j}^-)^2 }$$
5. Calculate the closeness of each alternative solution to the ideal solution. $V_i = \frac{S_i^-}{S_i^- + S_i^+}$
6. Determining the rank order of all alternatives on the basis of their relative closeness to the ideal solutions. The larger the $V_i$ is, the better the alternative solution $A_i$ is. The best alternative solution is the one with the largest closeness to the ideal solution.

### TOPSIS results and discussion

To rank the task schedule sequences produced by various schedulers, the TOPSIS method is employed. This method optimizes the selection of schedules according to the user’s prioritized objectives, which include Turnaround Time, Resource Utilization, Communication Price, and Execution Price, in terms of their desirability. Tables 28, 29, and 30 presents the result of the TOPSIS algorithm when applied to standard, random, and scientific task graphs, respectively. We explore different possible priority orders that users may assign to each criterion. Notably, we find a consistent ranking pattern for schedule sequences across all types of graphs, including Standard, Random, and Scientific graphs, which encompass Fully Connected, Pipeline, Star, Ternary, and Independent graph categories. Additionally, this ranking consistency persists even when the number of tasks varies (40, 121, 364, and 1039).

Weightage types 1, 2, 3, 4, as well as 7, 8, 9, and 10, exemplify situations where the user places the highest importance on turnaround time and resource utilization as criteria, while assigning less significance to communication cost and execution cost. In these scenarios, TOPSIS consistently ranks the greedy scheduler as the top solution. The second-best alternative solution is the greedy communication cost scheduler, which outperforms the other schedulers in terms of TAT and resource utilization.

However, in cases corresponding to weightage types 5, 6, 10, and 11, where the user’s preference primarily focuses on achieving an optimal communication cost, TOPSIS identifies the schedule generated by the greedy communication scheduler as the best solution. This scheduler minimizes communication costs to zero while maintaining TAT and resource utilization levels that are nearly on par with those achieved by the greedy scheduler. In this context, the output schedule sequence of the greedy scheduler is ranked last by TOPSIS, as it incurs the highest communication cost, contradicting the user’s prioritization of criteria desirability.

## Conclusion and future work

In this paper, we presented a multi-objective task scheduling framework for scheduling different types of workflows on computational grids. The main objective of our proposed framework is to minimize the overall execution cost, including application turnaround time and communication cost, while maximizing grid utilization. The proposed scheduling framework is integrated with GridSim and validated through experiments conducted on weighted standard task graphs, weighted random task graphs, and scientific task graphs. Furthermore, we envisaged a multi-criteria decision method called Technique for Order of Preference by Similarity to Ideal Solution (TOPSIS) to rank the output of the scheduling sequence based on different objective functions and the requirements of both users and service providers.

As part of future work, we plan to design a multi-objective task scheduling framework based on Large Language Models (LLMs) and compare the performance with NSGA-II in a computational cloud computing environment.

## Data Availability

The data that supports the findings of this study are available from the corresponding author on request.

## References

[1] Casanova, H. & Dongarra, J. Network enabled solvers for scientific computing using the NetSolve system. In Proc. of 3rd International Conference on Algorithms and Architectures for Parallel Processing, Melbourne, VIC, Australia, pp. 17-33 (1998).

[2] Goux, J.P., Kulkarni, S., Linderoth, J. & Yoder, M. An enabling framework for master_worker applications on the computational grid. In 9th IEEE Int. Symposium on High Performance Distributed Computing, HPDC’00 (2000).

[3] Chervenak A, Foster I, Kesselman C, Salisbury C, Tuecke S (2000). The data grid: Toward an architecture for the distributed management and analysis of large scientific datasets. J. Netw. Comput. Appl..

[4] Beynon, M. D., Sussman, A., Catalyurek, U., Kurc, T. & Saltz, J. *Performance optimization for data intensive grid applications*. In Proc. Third Annual International Workshop on Active Middleware Services, USA, 97–105 (2001).

[5] Linderoth L, Wright SJ (2003). Decomposition algorithms for stochastic programming on a computational grid. Comput. Optim. Appl..

[6] Newman HB, Ellisman MH, Orcutt JA (2003). Data-intensive e-Science frontier research. Commun. ACM.

[7] Buyya R, Abramson D, Venugopal S (2005). The grid economy. Proc. IEEE.

[8] Paniagua, C., Xhafa, F., Caballé, S. & Daradoumis, T. A parallel grid-based implementation for real time processing of event log data in collaborative applications. In Parallel and Distributed Processing Techniques, PDPT2005, Las Vegas, USA, pp. 1177–1183 (2005).

[9] Arbona A (2007). A service-oriented grid infrastructure for biomedical data and compute services. IEEE Trans. NanoBiosci..

[10] Alonso JM, Ferrero JM, Hernandez V, Molto G, Saiz J, Trenor B (2008). A grid computing-based approach for the acceleration of simulations in cardiology. IEEE Trans. Inf. Technol. Biomed..

[11] Mishra, Manoj Kumar, Patel, Yashwant Singh, Rout, Yajnaseni & Mund, G.B. A survey on scheduling heuristics in grid computing environment, I.J. Modern Education and Computer Science, pp. 57-83 (2014).

[12] Tsai C, Rodrigues J (2014). Meta heuristic scheduling for cloud: A survey. IEEE Syst. J..

[13] Zhou Zhou, Zhigang Hu (2014). Task scheduling algorithm based on greedy strategy in cloud computing. Open Cybern. Syst. J..

[14] Kong X, Lin C, Jiang Y, Yan W, Chu X (2011). Efficient dynamic task scheduling in virtualized data centers with fuzzy prediction. J. Netw. Comput. Appl..

[15] Sun W (2012). A game theoretic resource allocation model based on extended second price sealed auction in grid computing. J. Comput..

[16] Grover, R. & Chabbra, A. Bio-inspired optimization techniques for job scheduling in grid computing. In 2016 IEEE International Conference on Recent Trends in Electronics, Information & Communication Technology (RTEICT), pp. 1902-1906 (2016).

[17] Bagchi TP, Bagchi TP (1999). The nondominated sorting genetic algorithm: NSGA. Multiobjective Scheduling by Genetic Algorithms.

[18] Deb K, Pratap A, Agarwal S, Meyarivan T (2002). A fast and elitist multiobjective genetic algorithm: NSGA-II. IEEE Trans. Evolut. Comput..

[19] Coello Coello, C. A. & Lechuga, M. S. MOPSO: a proposal for multiple objective particle swarm optimization Proceedings of the 2002 Congress on Evolutionary Computation. CEC’02 (Cat. No.02TH8600), Honolulu, HI, USA, pp. 1051-1056 (2002).

[20] Li, H. and Landa-Silva, D., An Adaptive Evolutionary Multi-Objective Approach Based on Simulated Annealing, Evolutionary Computation, pp. 561-595, (2011). 10.1162/EVCO_a_00038 21417745

[21] Lopez-Ibanez M, Stutzle T (2012). The automatic design of multiobjective ant colony optimization algorithms. IEEE Trans. Evolut. Comput..

[22] Zhou Aimin, Bo-Yang Qu, Li Hui, Zhao Shi-Zheng (2011). Multiobjective evolutionary algorithms: A survey of the state of the art. Swarm Evolut. Comput..

[23] Yang S (2013). A grid-based evolutionary algorithm for many-objective optimization. IEEE Trans. Evolut. Comput..

[24] Zuo L, Shu L, Dong S, Zhu C, Hara T (2015). A multi-objective optimization scheduling method based on the ant colony algorithm in cloud computing. IEEE Access.

[25] Wang H, Jin Y, Yao X (2017). Diversity assessment in many-objective optimization. IEEE Trans. Cybern..

[26] Tian Y, Cheng R, Zhang X, Jin Y (2017). PlatEMO: A MATLAB platform for evolutionary multi-objective optimization. IEEE Comput. Intell. Mag..

[27] Lin Q, Liu S, Zhu Q, Tang C, Song R, Chen J, Coello Coello CA, Wong K-C, Zhang J (2018). Particle swarm optimization with a balanceable fitness estimation for many-objective optimization problems. IEEE Trans. Evol. Comput..

[28] Sadhukhan, Arindam, & Sivasubramani, S. Multi-objective load scheduling in a smart grid environment. In 20th National Power Systems Conference (NPSC), IEEE (2018).

[29] Singh, J. & Tiwari, R. *Multi-Objective Optimal Scheduling of Electric Vehicles in Distribution System, 20th National Power Systems Conference (NPSC)*, 1–6 (2018).

[30] Lin Q, Liu S, Wong K-C, Gong M, Coello Coello CA, Chen J, Zhang J (2019). A clustering-based evolutionary algorithm for many-objective optimization problems. IEEE Trans. Evol. Comput..

[31] Leiva J, Pardo RC, Aguado J (2019). Data analytics-based multi-objective particle swarm optimization for determination of congestion thresholds in lv networks. Energies.

[32] Yuping L (2019). Optimization of multi-objective virtual machine based on ant colony intelligent algorithm. Int. J. Perform. Eng..

[33] Grewal, S. K. & Mangla, N. Deadline and Cost Optimization based Task Scheduling (DCOTS) in Cloud Computing Environment,4th International Conference on Intelligent Engineering and Management (ICIEM), London, United Kingdom, pp. 1-6 (2023).

[34] Cui Z, Zhao T, Wu L, Qin AK, Li J (2023). Multi-objective cloud task scheduling optimization based on evolutionary multi-factor algorithm. IEEE Trans. Cloud Comput..

[35] Shrichandran, G., Tinnaluri, V. S. N., Murugan, J. S., Meeradevi, T., Dwivedi, V. K. & Christal Mary, S. S. Hybrid Competitive Swarm Optimization Algorithm Based Scheduling in the Cloud Computing Environment. In 5th International Conference on Inventive Research in Computing Applications (ICIRCA), Coimbatore, India, pp. 1013-1018 (2023).

[36] Zhang H, Jia R (2023). Application of chaotic cat swarm optimization in cloud computing multi objective task scheduling. IEEE Access.

[37] Lipsa S, Dash RK, Ivković N, Cengiz K (2023). Task scheduling in cloud computing: A priority-based heuristic approach. IEEE Access.

[38] Lou J, Tang Z, Zhang S, Jia W, Zhao W, Li J (2023). Cost-effective scheduling for dependent tasks with tight deadline constraints in mobile edge computing. IEEE Trans. Mobile Comput..

[39] Ajinkya Wagaskar, K. & Chowdhary, G. V. Optimal Resource Search in Grid Computing as a Multi-Objective Problem with Particle Swarm Technique. In International Conference for Emerging Technology (INCET), Belgaum, India, pp. 1-6 (2020).

[40] Alsadie D (2021). TSMGWO: Optimizing task schedule using multi-objectives grey wolf optimizer for cloud data centers. IEEE Access.

[41] Ni L, Sun X, Li X, Zhang J (2021). Gcwoas2: Multiobjective task scheduling strategy based on gaussian cloud-whale optimization in cloud computing. Comput. Intell. Neurosci..

[42] Abualigah L, Diabat A (2021). A novel hybrid antlion optimization algorithm for multiobjective task scheduling problems in cloud computing environments. Clust. Comput..

[43] Dutta Debashis (2022). Subhabrata rath job scheduling on computational grids using multi-objective fuzzy particle swarm optimization. Soft Comput. Theor. Appl..

[44] Kaur K, Garg S, Aujla GS, Kumar N, Zomaya AY (2022). A multi-objective optimization scheme for job scheduling in sustainable cloud data centers. IEEE Trans. Cloud Comput..

[45] Akbar MI, Kazmi SAA, Alrumayh O, Khan ZA, Altamimi A, Malik MM (2022). A novel hybrid optimization-based algorithm for the single and multi-objective achievement with optimal DG allocations in distribution networks’. IEEE Access.

[46] Moazeni A, Khorsand R, Ramezanpour M (2023). dynamic resource allocation using an adaptive multi-objective teaching-learning based optimization algorithm in cloud. IEEE Access.

[47] Reddy BPV, Reddy KG (2023). A multi-objective based scheduling framework for effective resource utilization in cloud computing. IEEE Access.

[48] Dakkak O, Suki A, Arif M, Shahrudin AN (2015). A critical analysis of simulators in grid. J. Teknol..

[49] Wu, R., Wu, M., Mi, X. & An, Q. Task Scheduling Algorithm Based on Triangle Module in Grid Computing. In 8th International Conference on Wireless Communications, Networking and Mobile Computing, 2012, pp. 1-4 (2012).

[50] Patel DK, Tripathy CR (2016). An efficient load balancing mechanism with cost estimation on GridSim. Int. Conf. Inf. Technol. (ICIT).

[51] Eng K, Muhammed A, Mohamed MA, Hasan S (2017). Incorporating the range-based method into GridSim for modeling task and resource heterogeneity. IEEE Access.

[52] Nukarapu D, Tang B, Wang L, Lu S (2011). Data replication in data intensive scientific applications with [32]performance guarantee. IEEE Trans. Parallel Distrib. Syst..

[53] Haider S, Nazir B (2017). Dynamic and adaptive fault tolerant scheduling with QoS consideration in computational grid. IEEE Access.

[54] Patel, D. K. & Tripathy, C. R. An Effective Selection Method for Scheduling of Gridlets among Heterogeneous Resources with Load Balancing on GridSim. In 2017 3rd International Conference on Computational Intelligence and Networks (CINE), pp. 68-72 (2017).

[55] Sheikh, S., Shahid, M. & Nagaraju, A. “A novel dynamic task scheduling strategy for computational grid. In 2017 International Conference on Intelligent Communication and Computational Techniques (ICCT), 2017, pp. 102-107 (2017).

[56] Hwang CL, Yoon K (1981). Multiple Attribute Decision Making: Methods and Applications.

[57] Yoon K (1987). A reconciliation among discrete compromise situations. J. Oper. Res. Soc..

[58] Hwang CL, Lai YJ, Liu TY (1993). A new approach for multiple objective decision making. Comput. Oper. Res..

[59] Krohling RA, Pacheco AGC (2015). A-TOPSIS - An approach based on TOPSIS for ranking evolutionary algorithms. Procedia Comput. Sci..

[60] Fei Liguo, Yong Hu, Xiao Fuyuan, Chen Luyuan, Deng Yong (2016). Modified TOPSIS method based on numbers and its applications in human resources selection. Math. Probl. Eng..

[61] Shirvani, M. H., Amirsoleimani, N., Salimpour, S. & Azab, A. Multi-criteria task scheduling in distributed systems based on fuzzy TOPSIS. In IEEE 30th Canadian Conference on Electrical and Computer Engineering (CCECE), pp. 1-4 (2017).

[62] Liu L, Fan Q, Buyya R (2018). A deadline-constrained multi-objective task scheduling algorithm in mobile cloud environments. IEEE Access.

[63] Srinivas, D.B., Hegde, S. N., Rajan, M. A. & Krishnappa, H. K. A Novel Task Scheduling Scheme for Computational Grids - Greedy Approach. In 2018 IEEE 32nd International Conference on Advanced Information Networking and Applications (AINA), 2018, pp. 1026-1033 (2018).

[64] Srinivas DB, Hegde Sujay N, Rajan MA, Krishnappa HK (2020). An efficient greedy task scheduling algorithm for heterogeneous inter-dependent tasks on computational grids. Int. J. Grid Util. Comput..

[65] Pegasus workflow generator: https://confluence.pegasus.isi.edu.

[66] Buyya Rajkumar, Murshed Manzur (2002). Gridsim: A toolkit for the modeling and simulation of distributed resource management and scheduling for grid computing. Concurr. Comput. Pract. Exp..

